# Under-ice observations by trawls and multi-frequency acoustics in the Central Arctic Ocean reveals abundance and composition of pelagic fauna

**DOI:** 10.1038/s41598-023-27957-x

**Published:** 2023-01-18

**Authors:** Randi B. Ingvaldsen, Elena Eriksen, Harald Gjøsæter, Arill Engås, Birte Katarina Schuppe, Karen M. Assmann, Heather Cannaby, Padmini Dalpadado, Bodil A. Bluhm

**Affiliations:** 1 Institute of Marine Research, Nordnes, B.O. Box 1870, 5817 Bergen, Norway; 2 Institute of Marine Research, Framsenteret, Stakkevollan, Postboks 6606, 9296 Tromsö, Norway; 3 UIT The Arctic University of Norway, Hansine Hansens veg 18, 9037 Tromsö, Norway

**Keywords:** Biophysics, Ecology, Climate sciences, Ecology, Environmental sciences

## Abstract

The rapid ongoing changes in the Central Arctic Ocean call for baseline information on the pelagic fauna. However, sampling for motile organisms which easily escape vertically towed nets is challenging. Here, we report the species composition and catch weight of pelagic fishes and larger zooplankton from 12 trawl hauls conducted in ice covered waters in the Central Arctic Ocean beyond the continental slopes in late summer. Combined trawl catches with acoustics data revealed low amounts of fish and zooplankton from the advective influenced slope region in the Nansen Basin in the south to the ice-covered deep Amundsen Basin in the north. Both arctic and subarctic-boreal species, including the ones considered as Atlantic expatriate species were found all the way to 87.5^o ^N. We found three fish species (*Boreogadus saida, Benthosema glaciale* and *Reinhardtius hippoglossoides*), but the catch was limited to only seven individuals. Euphausiids, amphipods and gelatinous zooplankton dominated the catch weight in the Nansen Basin in the mesopelagic communities. Euphausiids were almost absent in the Amundsen Basin with copepods, amphipods, chaetognaths and gelatinous zooplankton dominating. We postulate asymmetric conditions in the pelagic ecosystems of the western and eastern Eurasian Basin caused by ice and ocean circulation regimes.

## Introduction

The Central Arctic Ocean (CAO) becomes increasingly ice-free during the summer due to climate warming^1^. Oceanographic changes are also underway, namely amplified warming and increasing salinity in the upper waters of the Eurasian Basin related to upstream conditions^2^. This will eventually affect the whole ecosystem in various ways^3,4^, and dramatic changes are expected in terms of magnitude and spatial distribution of ice-associated and pelagic primary production^5–7^. To monitor these changes, it is urgent to establish a baseline mapping of an understudied area in the central Arctic. This is particularly relevant for species potentially subjected to future fisheries^8^.

Earlier expeditions to ice-covered waters in the Eurasian Basin have begun to map biodiversity and faunal densities/biomass patterns. They revealed that polar cod (*Boreogadus saida*) and various ice-associated and pelagic phytoplankton and zooplankton species are present at the underside of the ice and in the upper parts of the water column below the ice^5,9–12^. Vertical zonation of mesozooplankton in the Eurasian Basin has also been characterized and synthesized over the past decades^11,12^. Some larger organisms, like armhook squid (*Gonatus fabricii*) and gelatinous zooplankton and nekton have been found^11,13^, as well as single individuals of Atlantic cod (*Gadus morhua*), glacier lanternfish (*Benthosema glaciale)* and ice cod *(Arctogadus glacialis)*^13^. Faunal densities and biomass for the mapped Eurasian Basin water column are generally low in comparison to Arctic shelves and southern areas^11,12,14^ due to overall low primary production and hence food availability^15,16^. However, the presence and distribution of larger and motile organisms like fish and macrozooplankton have been poorly described in the CAO due to severe difficulties in sampling such species in ice covered areas.

Scattering layers in the mesopelagic (200–1000 m) zone are ubiquitous in most world oceans^17,18^. Several studies conducted around the perimeter of the CAO have also demonstrated that deep scattering layers exist in these regions^19–22^. Recent studies confirm that such a deep scattering layer is present both in the Eurasian and Amerasian ice-covered basins, but with much lower backscatter energy compared to e.g., the Norwegian Sea^21,23^. Knowledge on the links between oceanographic regimes and mesopelagic biomass and biodiversity is needed to predict how these organisms will be impacted under climate change^24^. The present study partly fills these gaps. Our hypotheses were that pelagic fishes and large zooplankton in the CAO (1) occur in lower abundances compared to regions further south, and (2) in their taxonomic composition reflect recent physical changes in that subarctic-boreal species present also penetrate the northernmost parts of the Eurasian Basin.

**Figure 1:**
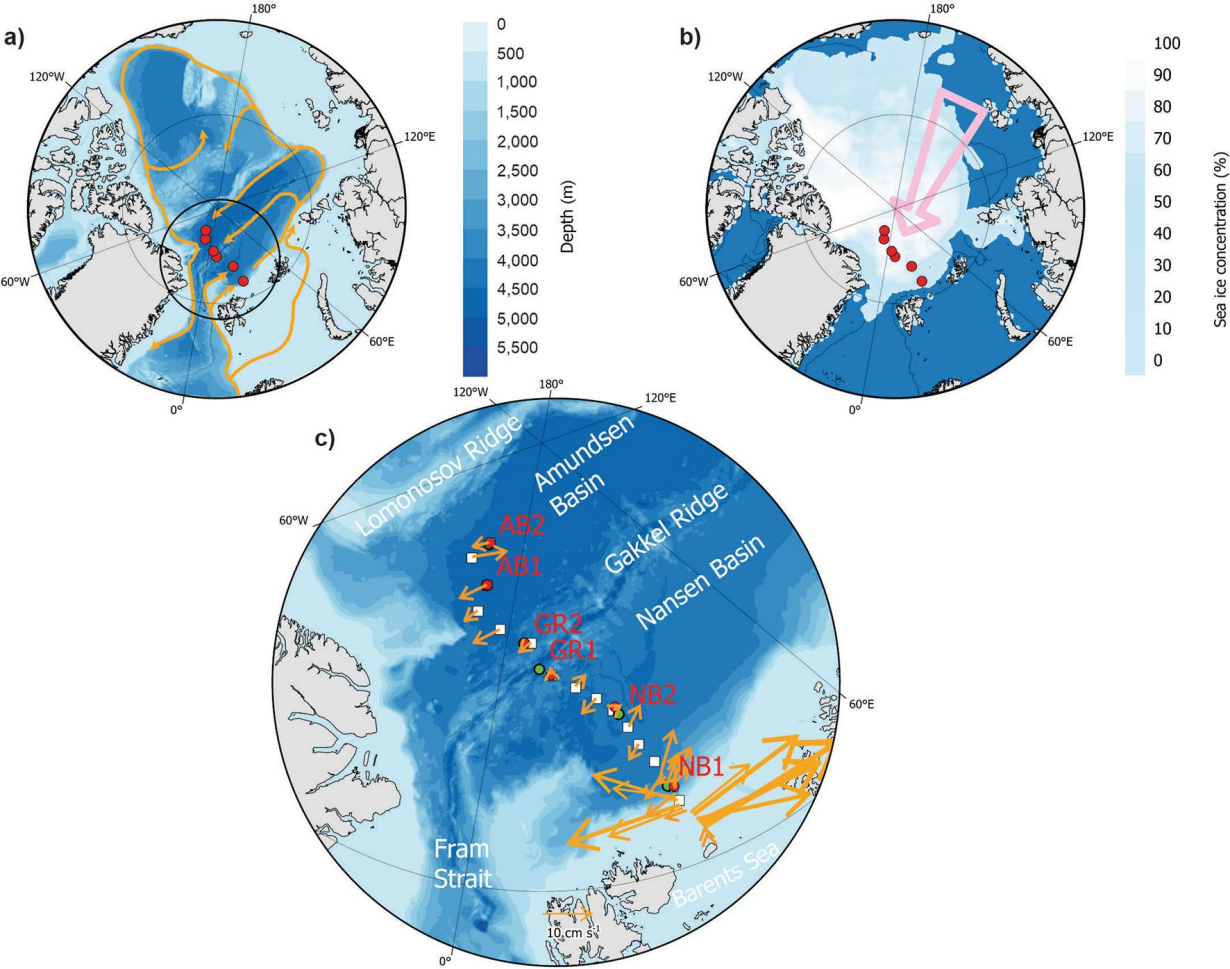
Pelagic trawl stations and physical conditions. Map of the Arctic Ocean (**a**) with trawl locations () in the Eurasian Basin in September 2021. Flow of Atlantic Water^26^ is shown with  and  denote the region shown in (**c**). Mean sea ice concentration during the survey (**b**). The pink arrow denotes the Transpolar Drift^27^ and  shows the 500 m bottom contour. Average 25–250 m depth ocean currents () in the survey (**c**).  show locations where acoustic data have been scrutinised and  show CTD stations. The maps were made using ArcGIS Pro 2.9.5 (https://www.esri.com/en-us/arcgis/products/arcgis-pro/overview).

In August–September 2021 the Nansen Legacy Joint Cruise 2-2 (JC2-2) with R/V *Kronprins Haakon* covered a transect of ca. 2330 km extending from the Nansen Basin northeast of the Svalbard slope, to 87.5^o ^N at the northern side of the Amundsen Basin just south of the Lomonosov Ridge^25^ (Fig. 1a). The region is characterized by heavy sea ice (concentrations spanning 30–90% during the survey, Fig. 1b), and strong inflow of Atlantic Water along the slope north of Svalbard with weaker flow in the Nansen and Amundsen Basins (Fig. 1a, c). To test our hypotheses, we sampled six locations with pelagic trawls modified for operation in ice-covered waters (Fig. 1), covering both the epipelagic (< 200 m) and the mesopelagic layers. This included one location slightly north of the slope in the Nansen Basin (NB1) and one location in the central parts of the Nansen Basin (NB2), two locations on the southern and northern side of the Gakkel Ridge (GR1 and GR2) and two locations in the deep Amundsen Basin (AB1 and AB2). Additionally, echo soundings at six frequencies were recorded at the locations (Fig. 1c). We report for the first time on catches taken by ice-modified trawls in the epi- and mesopelagic layers and document how these, in combination with multi-frequency acoustic records, give baseline information on the ecological aspects in ice-covered parts of the CAO.

## Results

### Sea ice and oceanic conditions.

**Figure 2:**
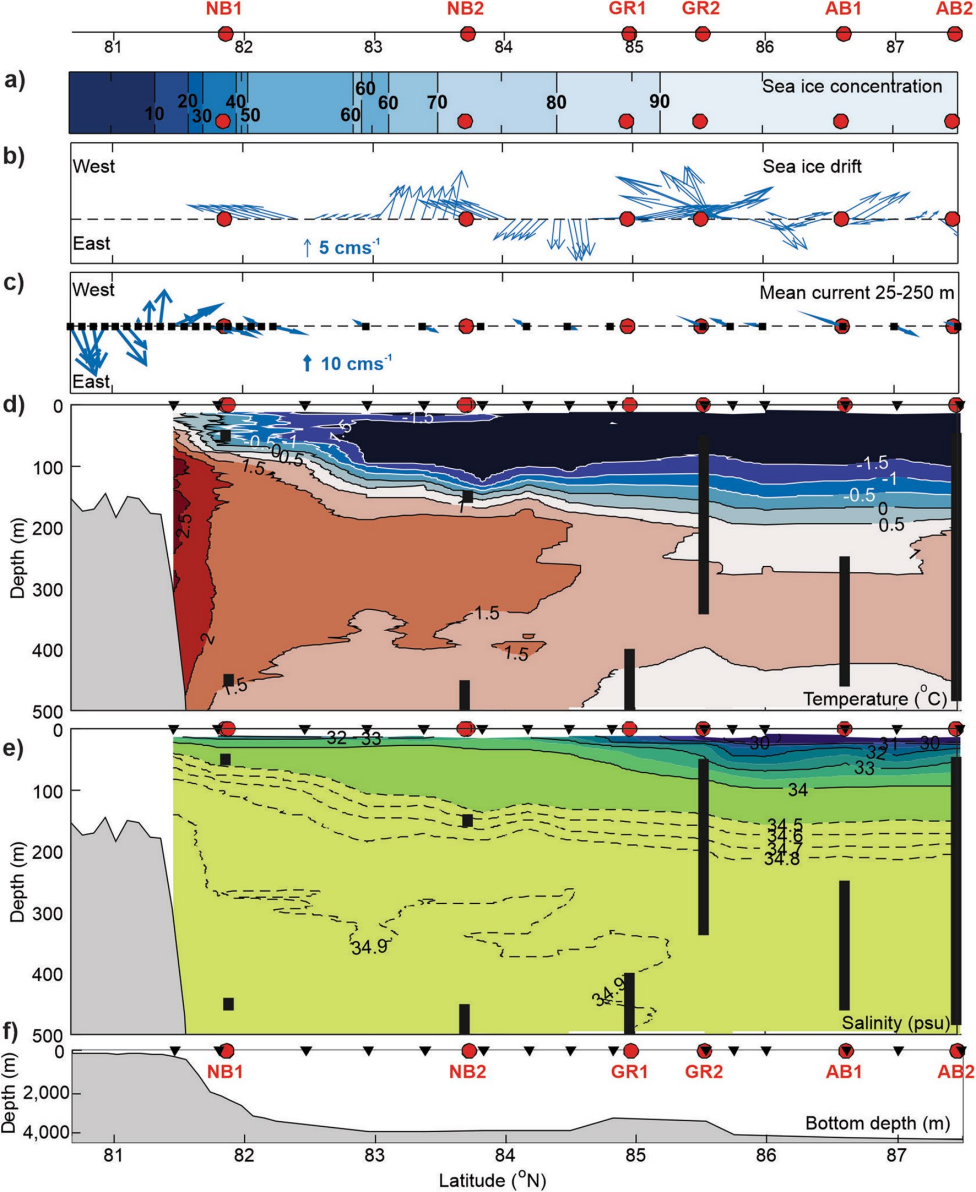
Environmental conditions in the Eurasian Basin in September 2021. Mean sea ice concentration during the survey (**a**) and sea ice drift at the time of sampling (**b**). Mean ocean velocity in the 25–250 m depth layer (**c**), temperature (**d**) and salinity (**e**) in the upper 500 m. The part of the water column covered by the trawl hauls (Harstad or macroplankton or both) are shown with  (in d and e) and bathymetry is shown in (**f**). The location of the trawl locations () and CTD stations () are shown in all panels.

The trawl and acoustic sampling were conducted in a region spanning strong gradients in sea ice and oceanic conditions (Fig. 2). The sea ice concentration increased substantially just to the north of NB1, and NB2 and GR1 had sea ice concentrations between 70 and 90% (Fig. 2a). Sea ice concentration at the three northernmost locations exceeded 90%. The sea ice drift during sampling was only modest with mean drift mostly from east (northeast or southeast) to west except at location AB2 and between location NB2 and GR1 when it was reversed (Fig. 2b).

Velocity, temperature, and salinity also changed rapidly to the north of NB1 (Fig. 2c–e). From about 83^o ^N, extremely cold (− 1.8 °C < T < − 1.5 °C) and fresh polar waters covered the upper 100 m. A pronounced surface salinity front appeared in the northern Nansen Basin-Gakkel Ridge region (Fig. 2e), and the vertical stratification strengthened going northwards due to decreasing upper layer salinities. Warmer water (T > 0 °C) of Atlantic origin was present below 125–170 m depth (Fig. 2d).

The vessel-mounted ADCP data revealed eastwards velocities reaching 25–30 cms^-1^ along the slope of the Nansen Basin (Figs. 1c and 2c). In this region, warm and saline Atlantic Water which enters through Fram Strait (Fig. 1a) flows eastwards into the Arctic Ocean^28^. Our southernmost location NB1 was slightly to the north of the slope and main core (located between the 300 and 1000 m isobaths^29,30^) of the Atlantic Water current. Instead, the ADCP data showed rapidly varying horizontal flow near NB1 indicating mesoscale eddies (Figs. 2c and S1). To the north of NB1 (Figs. 1c and 2c), our mean 25–250 m ocean currents showed mostly weak, zonal flow, i.e., no direct flow from the southern part of the transect to the northern parts.

### Organisms caught by trawls

Twelve trawl hauls were taken in pairs at the six locations along the route: eight hauls with a Harstad trawl targeting pelagic fish and four hauls with a macroplankton trawl for catching larger zooplankton (Table 1). Three hauls were towed in the epipelagic layer, six hauls in mesopelagic layers and three covering both layers.

**Table 1:** Sampling effort with the trawls in the Arctic Basins.

Station code	Station no	NL station	Date (2021)	Latitude	Longitude	Fishing depth (m)	Trawl opening height (m)	Trawl door spread (m)
NB1e	3601	P7b	28.08	81.86	30.15	60–40	12.37 (± 1.09)	44.68 (± 2.33)
NB1m	3602	P7b	28.08	81.88	30.21	460–450	9.73 (± 0.78)	71.46 (± 3.08)
NB2e	3603	P8a	31.08	83.72	25.87	160–150	11.69 (± 1.6)	61.71 (± 2.97)
NB2m	3604	P8a	01.09	83.69	25.97	600–450	10.35 (± 1.54)	71.04 (± 3.32)
GR1m	3605	P9a	05.09	84.96	9.42	550–400	12.29 (± 2.27)	46.8 (± 13.64)
GR1mMT	3606	P9a	05.09	84.97	9.51	537–390		35.23 (± 4.27)
GR2e	3607	P9	08.09	85.52	4.99	211–50	11.53 (± 1.52)	56.37 (± 6.79)
GR2emMT	3608	P9	08.09	85.51	4.63	347–60		40.93 (± 7.6)
AB1m	3611	NLEG39	19.09	86.60	− 11.109	460–300		68.96 (± 3.31)
AB1mMT	3612	NLEG39	19.09	86.63	− 11.19	430–260		29.99 (± 3.98)
AB2em	3609	P11a	15.09	87.45	− 17.419	46–484–46	9.63 (± 3.64)	68.99 (± 6.8)
AB2emMT	3610	P11a	15.09	87.46	− 17.40	49–483–49		32.95 (± 5.17)

Sampling information includes fish station code, station number, Nansen Legacy project station name, date, location, fishing depth, mean and standard deviation for opening height (m) and door spread (m). Station code includes labelling for areas where samples were taken: Nansen Basin (NB), Gakkel Ridge (GR) and Amundsen Basin (AB). The station code also includes a notation denoting the depth of the trawl (e, epipelagic; m, mesopelagic, and em, both layers). The trawls denoted with MT were macroplankton trawls, while the others were Harstad trawls. Additional information, including on the acoustic stations and the CTDs, can be found in Table S1.

**Figure 3:**
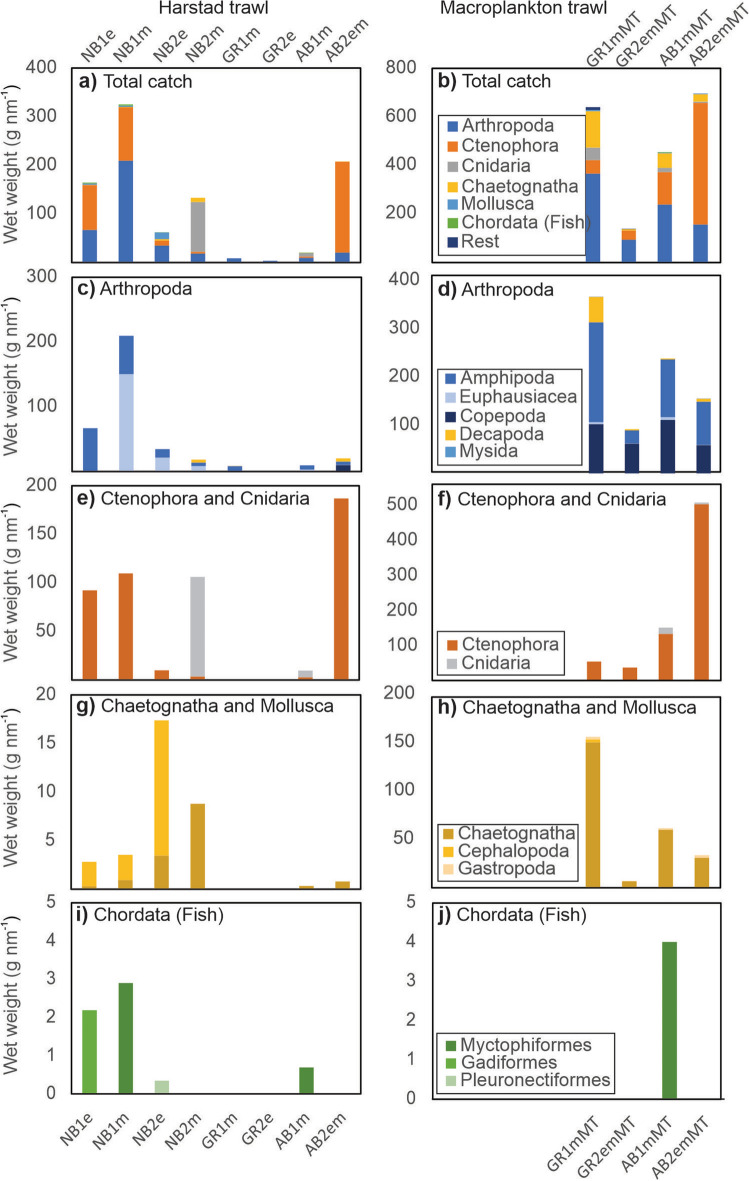
Composition of pelagic trawl catches in the Eurasian Basin. Left panels show catches from the Harstad trawl (**a, c, e, g, i**) and right panels show catches from the macroplankton trawl (**b, d, f, h, j**) in September 2021.

The biological samples contained 26 taxa, with some organisms identified to species level, and others to family, order, or phylum levels (Table 2). The trawl catches by the macroplankton and Harstad trawls confirmed the hypothesized ubiquitous occurrence but generally low abundance of organisms, including fish, in the CAO. All Harstad trawl catches were very small and varied between 3 and 325 g nm^−1^ (Fig. 3a, Table S2). The two first hauls in the Nansen Basin had the largest catches (NB1e and NB1m, 164 g nm^−1^ and 325 g nm^−1^ respectively), together with one haul in the Amundsen Basin (AB2em, 208 g nm^−1^). The Harstad trawl revealed very small catches at the Gakkel Ridge (GR1m and GR2m, 9 g nm^−1^ and 3 g nm^−1^ respectively). The four hauls with the finer-meshed macroplankton trawl generally yielded higher catches (137–696 g nm^−1^) than the Harstad trawl (Fig. 3b, Table S2).

**Table 2:** Taxa recorded during the survey.

Phylum	Family	Taxon/species name	Biogeographical classification	Location
Arthropoda	Calanidae	*Calanus* spp.		NB2, AB1, AB2
Arthropoda	Calanidae	*Calanus hyperboreus*	Arctic^11,12^	***GR1, GR2, AB1, AB2***
Arthropoda	Euchaetidae	*Paraeuchaeta spp. (P. glacialis & P. barbata)*	Boreal-Arctic & cosmopolitan^37,38^	***GR1, GR2, AB1, AB2***
Arthropoda	Augaptilidae	*Euaugaptilus* sp. *(E. hyperboreus)*	Arctic^39^	***GR1***
Arthropoda	Aetididae	*Pseudochirella* spp. *(P. batillipa; P. spectabilis)*	Subarctic & Arctic^11,40^	***GR1***
Arthropoda	Mysidae	Mysidae indet		***GR1, AB1***
Arthropoda	Euphausiidae	*Meganyctiphanes norvegica*	Atlantic boreal^41^	NB1, NB2, ***GR1***, AB1, AB2
Arthropoda	Euphausiidae	*Thysanoessa inermis*	Boreal & Subarctic^41,42^	NB2, ***GR2, AB1, AB2***
Arthropoda	Euphausiidae	*Thysanoessa longicaudata*	Boreal & Subarctic^41,42^	***AB1, AB2***
Arthropoda	Euphausiidae	***Nematoscelis megalops***	Subtropical-Boreal^41^	***GR1***
Arthropoda	Hyperiidae	*Themisto abyssorum*	Boreal & Subarctic^39,43,44^	NB2, ***GR2***, AB1, ***AB2***
Arthropoda	Hyperiidae	*Themisto libellula*	Arctic^38,43,44^	All locations
Arthropoda	Eusiridae	*Eusirus holmii*	Arctic^45^	***GR1, AB1***, AB2
Arthropoda	Lysianassidae	*Cyclocaris guilelmi*	Arctic^11,46^	***GR1***
Arthropoda	Acanthephyridae	*Hymenodora glacialis*	Arctic^11,47^	NB2, GR1, GR2***, AM1***, AM2
Arthropoda		*Caridea* indet		***GR1***
Ctenophora	Beroidae	*Beroe cucumis*	Boreal & Subarctic^11,19^	NB1, NB2, ***GR1, GR2***, AM1, AM2
Ctenophora	Mertensiidae	*Mertensia* spp.	Boreal & Subarctic^11,19^	***GR1, GR2, AB1***
Cnidaria		Hydroidolina		AB1, ***AB2***
Cnidaria	Periphyllidae	***Periphylla periphylla***	Boreal & Subarctic^48^	NB2
Cnidaria		Indet		***GR1, AB1, AB2***
Chaetognatha		Chaetognatha (*Pseudosagitta maxima, Parasagitta elegans, Eukrohnia hamata*)	Boreal & Subarctic^11,49^	NB1, NB2, ***GR1, GR2***, AM1, AM2
Mollusca	Clionidae	*Clione limacina*	Boreal & Subarctic^11,19^	***GR1, GR2, AB1, AB2***
Mollusca	Gonatidae	*Gonatus* sp.	Boreal^19^	NB1, NB2, ***GR1***
Chordata	Myctophidae	*Benthosema glaciale*	Boreal & Arctic^19^	NB1, AB1
Chordata	Gadidae	*Boreogadus saida*	Arctic^19^	NB1
Chordata	Pleuronectidae	***Reinhardtius hippoglossoides***	Boreal & Arctic^19^	NB2

Phylum, family and species/taxon names are given with nomenclature consistent with the World Register of Marine Species. In bold print are species for which our study provided the first record from the study area from our knowledge. Biogeographical affinity and sampling location are also shown. Locations where the taxa were sampled only by the macroplankton trawl are shown in bold and italics font. Photographs of the catches are given in Figs. S3–S14.

The Harstad trawl caught a total of 15 taxa, namely included Ctenophora (44%), Arthropoda (40%), Cnidaria (12%), Chaetognatha (2%), Mollusca (2%) and Teleostei (1%) by wet weight (Fig. 3a). Euphausiids (*Meganyctiphanes norvegica,* 3.6–4.0 cm, Table S3), amphipods (*Themisto abyssorum*, 1.2–1.4 cm, and *T. libellula*, 2.1–2.6 cm, Table S3) and periphyllids (*Periphylla periphylla*) contributed most to the catches in the Nansen Basin, while *T. libellula* (3.0–3.8 cm, Table S3) dominated the catches in the Amundsen Basin and at the Gakkel Ridge (Fig. 3). For *P. periphylla*, our record is the furthest north to our knowledge. Three fish families, each represented by one species, were found including Myctophidae (glacier lanternfish), Gadidae (polar cod), and Pleuronectidae (Greenland halibut *Reinhardtius hippoglossoides*). The few fish sampled included one 8 cm (total length) polar cod (at NB1e), one 4 cm Greenland halibut larva (at NB2e), three glacier lanternfishes at NB1m (4.5, 4.5 and 5.5 cm) and one at AB1m (5.0 cm). This is, as far as we know, the first time glacier lanternfish has been caught this far north, although the species has been tentatively identified by video recordings even further to the north in this region^13^. It is also to our knowledge the northernmost record of Greenland halibut, and the first time it has been observed north of the shelf break in the CAO.

The macroplankton trawl had higher taxon richness (23) and catches consisted of Arthropoda (44%), Ctenophora (38%), Chaetognatha (13%), Cnidaria (4%), Mollusca (0.6%) and Teleostei (0.2%) by wet weight (Fig. 3b). In the phylum Arthropoda, the copepod *Calanus hyperboreus* and the amphipod *Themisto libellula* (2.3–2.6 cm, Table S3) dominated the catch, whereas *Beroe cucumis* and *Mertensia* spp. contributed to Ctenophora biomass (Table S2). In addition, chaetognaths (*Pseudosagitta maxima*, *P. elegans* and *Eukrohnia hamata*) were found. Notably, more than 90% of the *Calanus hyperboreus* and *Paraeuchaeta* spp. recorded were mature females (Fig. S15). Biomass of euphausiids (*Thysanoessa inermis*, 1.7–2.4 cm, *T. longicaudata,* 1.4 cm, and other species) was also low in the macroplankton trawl catches. Though their biomass was comparatively low, the warm water associated species such as *Nematoscelis megalops* and *Meganyctiphanes norvegica* were recorded reaching far north in the Arctic waters. In fact, this is the first record of *N. megalops* in the study area as far as we know. The macroplankton trawl also caught a single glacier lanternfish (6.5 cm) at AB1.

As expected, the macroplankton trawl had higher catches of the smaller organisms (e.g., copepods, ctenophores, and chaetognaths) compared to the Harstad trawl (Fig. 3). Twelve plankton taxa were caught by the macroplankton trawl only (Table 2). Combining results from both trawls catches show that some taxa such as Ctenophora (*Beroe cucumis)* and hyperiid amphipods (*Themisto libellula*) were observed along the whole transect. Copepoda is another group that we expect to be represented at all stations, however, this was not evident as the gears we used are not suitable for catching mesozooplankton. In the macroplankton trawl, though, we recorded biomass of the larger *Calanus hyperboreus* (ca. 7 mm), these are most likely underestimated. Other taxa were taken at either only southern or northern locations. The amphipod *Eusirus holmii* and the decapod *Hymenodora glacialis* were found at the northernmost locations only. Polar cod and Greenland halibut were caught at southern locations only, while glacier lanternfish occurred at both southern and northern locations.

### Multi-frequency acoustics

**Figure 4:**
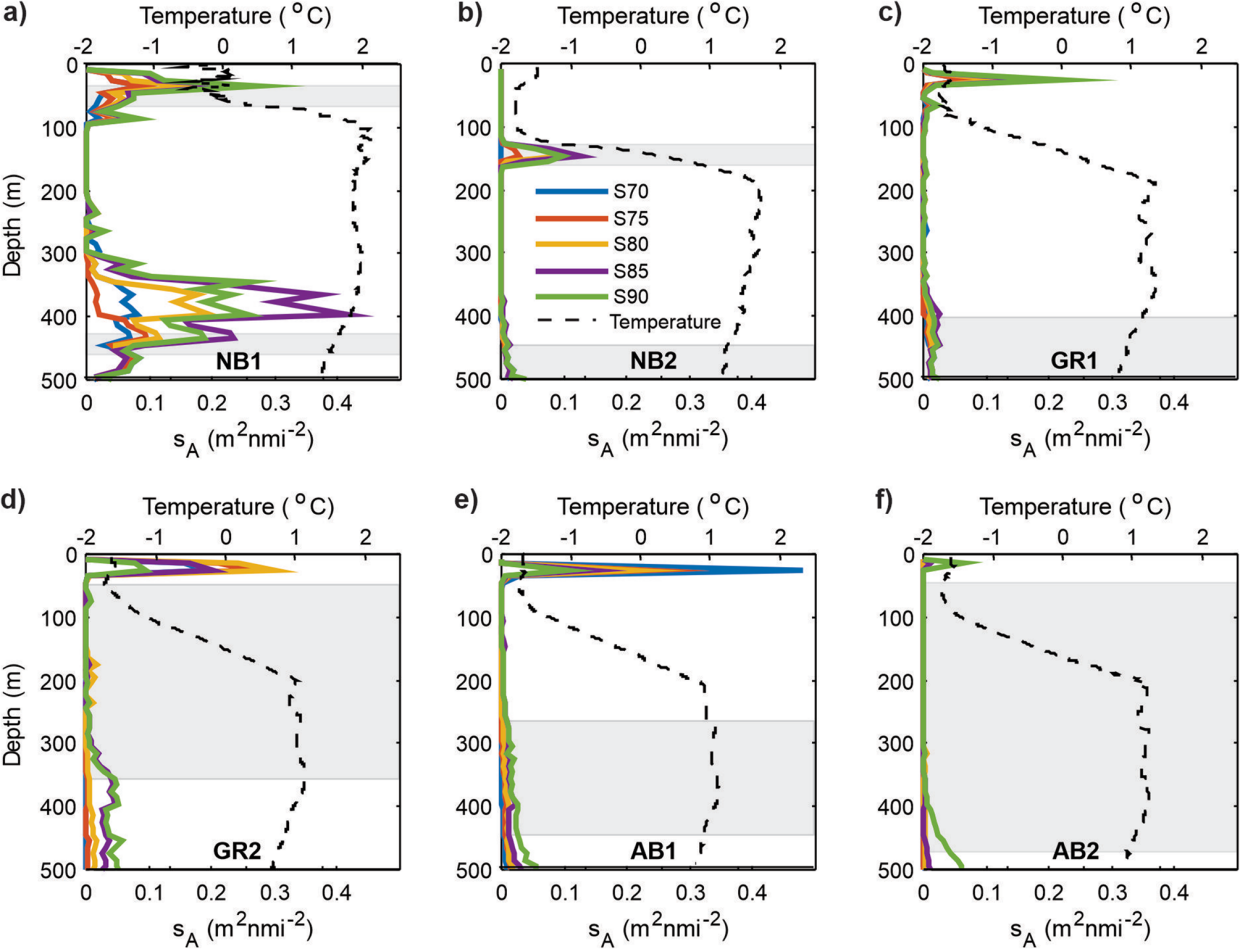
Acoustic backscatter in the Eurasian Basin. Profiles of acoustic backscatter from six locations in September 2021 (**a-f**). The various categories of scatterers are coded with colours. ---- show the temperature from the nearest CTD station, while  are the depth intervals where the trawls were conducted (Harstad or macroplankton or both). Note that a single strong echo, observed at about 265 m depth at NB1 (Fig. S18) was excluded when plotting this figure.

The acoustic data were categorized using sequential thresholding, frequency response (rf _18/38_), and target strength (TS) analysis (Supplementary section 3). The sequential thresholding showed epi- and mesopelagic layers at all locations (Fig. 4), albeit with varying strength and much weaker than usually observed in the Norwegian and Barents Seas. In the Nansen Basin, a faint (maximum s_A_ of 0.3–0.4 m^2^ nmi^-2^, where s_A_ is the Nautical area scattering coefficient (NASC)^50^) acoustic scattering layer was present at NB1 at 10–100 m in water with temperatures slightly below zero (Fig. 4a and S16). The layer contained mostly weak scatterers as captured with the S70 and S75 categories, and all organisms in this layer had target strengths at 38 kHz (TS_38_) weaker than − 55 dB (Fig. S17). The frequency response (rf_18/38_ = 4.0) suggested that organisms containing gas were present. The matching catch from the Harstad trawl (in 60–40 m) showed among other things one 8 cm long polar cod (Table S2). The mean TS_38_ of such a fish would be about − 54 dB^51^. No such TS_38_ detections were evident, and most acoustic targets had lower TS_38_ and were probably plankton organisms, which also totally dominated the trawl catches (Fig. 3, Table S2).

Compared to the other stations, a stronger mesopelagic scattering layer was present at NB1 in 300–500 m depth in Atlantic Water temperatures (1.5–2.0 °C) (Figs. 4a and S16). The TS analysis implied that the layer contained larger fish than caught by the trawl, like for instance, polar cod (Supplementary section 3). If so, the observed TS_38_ mode would translate into fishes of lengths of about 7 cm^51^. In addition, one single echo from a target at 265 m stood out with an s_A_ more than 10 times higher than the rest (Fig. S18). More than 20 TS_38_ detections of this target were in the range − 20 to − 32 dB.

In the central Nansen Basin (NB2), faint echoes forming a scattering layer could be seen in the depth interval 100–200 m (Figs. 4b and S19). Very cold polar water (− 1.8 °C < T < − 1.5 °C) occupied the upper 100 m and the scattering appeared at depths where the temperature approached 0 °C (Fig. 4b). All targets had TS_38_ weaker than − 50 dB, indicating that no larger fish were present. Consistently, the matching trawl catches consisted of a mixture of gelatinous and crustacean plankton and an armhook squid (Table S2). Additionally, one larval Greenland halibut (4 cm long) was caught, which would have a TS_38_ far lower than − 50 dB since it does not have swim bladder. The scattering in the mesopelagic layer was weak (Fig. 4b), but TS_38_ revealed a mode at − 52 dB (Fig. S17).

The echograms from the two locations at the Gakkel Ridge (GR1 and 2) showed a distinct epipelagic layer in 20–40 m depth, in polar waters with temperature below − 1.5 °C (Fig. 4c, d, Figs. S20, S21). A sharp peak of increased fluorescence in the upper water column overlapped with the increased acoustic scattering (Fig. S22), implying that the scattering stemmed from zooplankton gathered in a layer of phytoplankton. Consistently, the S75 and S80 categories at GR2 were higher than anywhere else in the transect (Fig. 4c, d), implying a different zooplankton composition or density. However, it is also possible that some of the backscatter originated from the sound velocity contrast in this pycnocline (Fig. S22). At greater depths there was a weak mesopelagic backscatter, with TS_38_ values with a mode for − 54 dB (Fig. S17). The frequency response (rf _18/38_ of 0.4–1.2) indicated a mixture of organisms with and without gas inclusions, while the catches from the Harstad (50–550 m) and macroplankton (60–537 m) trawls were dominated by arctic hyperiid amphipods (Fig. 3, Table S2).

In the southern Amundsen Basin (AB1), the 25–30 m depth layer had a stronger acoustic signal than elsewhere in the study area, and with a different composition and/or density (a stronger S70 fraction, Fig. 4e). The echo registrations at deeper water were weak, consistent with the trawls catches being dominated by a mixture of amphipods, copepods, ctenophores and chaetognaths with a clear dominance of smaller organisms (Fig. 3 and Table S2). However, the TS_38_ registrations at depth resembled those at the more southern locations, although with a wider distribution peaking at − 58 dB (Fig. S17). Nevertheless, also at this location the TS_38_ distribution at depth revealed numerous TS_38_ registrations at − 54 dB. In the central Amundsen Basin (AB2), both the epi- and mesopelagic scattering was weak (Figs. 4f and S17).

## Discussion

### Epi- and mesopelagic fauna in the western Nansen and Amundsen Basins

**Figure 5:**
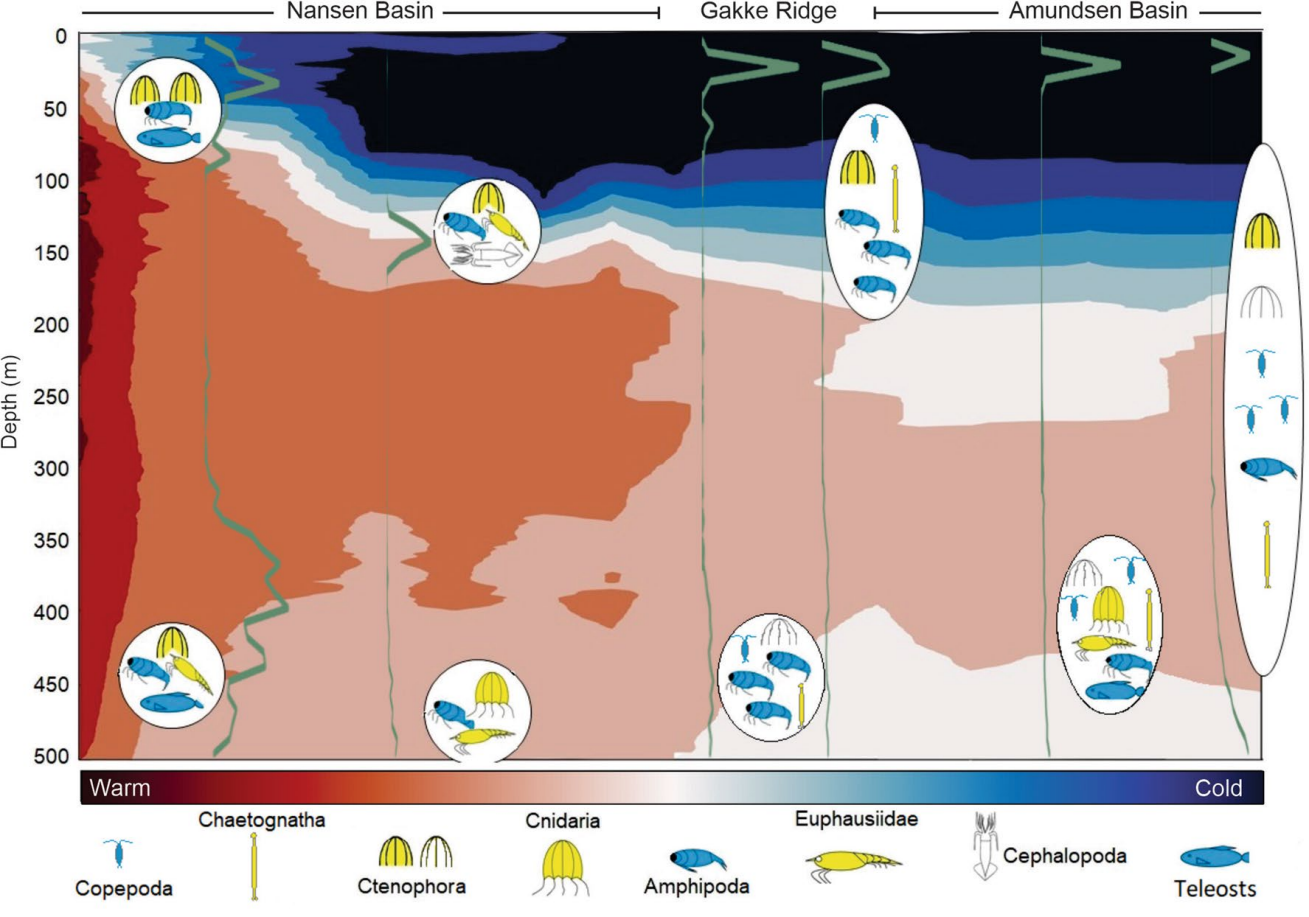
Schematics of dominant epi- and mesopelagic fauna along the Eurasian Basin transect based on trawl catches. Arctic organisms are shown in blue colour and subarctic-boreal ones in yellow. Symbols for organisms defined to higher taxa level (which could include species belonging to different bio-geographic affinity) are shown in white. Bubbles with organisms are place at approximate range of sampling depth. Note that absence of smaller organisms like copepods and chaetognaths in the Nansen Basin is due to sampling bias, as the macroplankton trawl was not used there. The green lines show backscatter strength while the background colour is the temperature field from Fig. 2d.

The survey covered a gradient from an advective/mesoscale eddy regime strongly influenced by the Atlantic Water slope current in the southern Nansen Basin towards a gradually more pronounced Arctic regime from ~ 83^o ^N and northwards (Fig. 2). However, also the Arctic regime had waters of Atlantic origin (T > 0 °C) at depth (Fig. 2d), holding a distinct mesopelagic scattering layer (Fig. 4), which has been shown to be widely distributed in the CAO^23^. Our trawl catches show that the mesopelagic layer contains both subarctic-boreal and Arctic species all the way north to 87.5^o ^N (Fig. 5), consistent with earlier results^11^. Some of the larger zooplankton species we found are also present in the Amerasian Basin as assessed by ROV^52^ while many of the smaller zooplankton species found there were not caught in our trawls, which likely is partly an effect of gear rather than biogeography.

### Near-slope in the Nansen Basin

Higher catches (Fig. 3) and acoustic backscattering (Fig. 4a) in the near-slope region in the Nansen Basin (NB1) compared to the locations visited further north are consistent with the high inflow of zooplankton in the Atlantic Water flow at the slope of the southern Nansen Basin^33,35,53–55^. Some advected taxa of boreal Atlantic origin, such as *Meganyctiphanes norvegica,* are not currently likely to reproduce in the Eurasian Basin^11^. However, if warming persists, these species may in future potentially complete their life cycles in these waters. These advective organisms play an important role as a substantial food source to consumers in the region^35^.

The epi- and mesopelagic fauna in the southern Nansen Basin was more diverse compared to further north (Fig. 5). It included well-documented subarctic-boreal ctenophores, both subarctic-boreal and arctic amphipods and euphausiids (consistent with a synthesis by Kosobokova^11^), and glacier lanternfish and polar cod. The acoustic data revealed a strong echo target (Fig. S18) with TS_38_ detections in a range implying a large (130–140 cm) Atlantic cod, or a marine mammal or a big fish without a swim bladder (Supplementary section 3). The most probable candidate is a Greenland shark (*Somniosus microcephalus),* which is commonly found near the slope in this area^56^. Juvenile Beaked redfish (*Sebastes mentella*), haddock (*Melanogrammus aeglefinus*), capelin (*Mallotus villosus*), polar cod, Atlantic cod and Greenland halibut can also be found in the meso- and epipelagic layers closer the slope slightly further west, yet some species appear to occur seasonally^19,21,57^. Also, adult redfish and Atlantic cod have been caught in the mesopelagic layer just off the slope slightly further west^21,58^. However, these reported catches were taken closer to the Atlantic Water slope current.

### The central Nansen Basin

Backscatter declines strongly in the upper mesopelagic zone when crossing boundaries between subpolar and polar water masses^59^, and such a decline was also evident when moving from the near-slope (NB1) to the central (NB2) Nansen Basin (Figs. 2 and 4a, b). It is worth nothing, however, that our observed gradient can be biased by dial migration occurring in these regions^20^. Since the central Nansen Basin location (NB2) was sampled near midnight (Table S1), the weak scattering at depth in combination with enhanced scattering near 150 m (Fig. 4b) at least partly can be caused by organisms, which have moved upwards from the deeper mesopelagic layer toward the surface during night-time.

The mesopelagic layer carried much the same species as near the slope (Fig. 5), with the addition of armhook squid and one larva of the subarctic-boreal Greenland halibut. Armhook squid appear to be widely distributed in the Arctic^13,21,52^. The Greenland halibut larva, on the other hand, has not been observed this far north before, and was likely advected with the Atlantic Water branch flowing around the Yermak Plateau (Fig. 1) or by mesoscale eddies during the summer. Both mesoscale eddies^30,31^ and recirculation of the Atlantic Water^32^ appear to be common in this region. Such smaller scale and regional features can provide a means for bringing subarctic-boreal organisms, advected with the Atlantic Water current^21,33–36^, off the slope and into the central Nansen Basin. Another possibility is that the Greenland halibut larva originates from the shelf-break on the Kara, Laptev Seas, or East Siberian Seas^60,61^.

The mesopelagic layer in the central Nansen Basin contained some crustacean plankton including subarctic-boreal euphausiids and arctic amphipods (Fig. 5), but was by weight dominated by the subarctic-boreal gelatinous deep-water plankton *Periphylla periphylla*. Increased inflow of Atlantic Water may have provided more suitable conditions for this species to colonise the European Arctic in recent decades^48^. Yet our record appears to be the furthest north so far.

### Gakkel Ridge

Despite heavy sea ice (80–90% concentration) and low (T < − 1.5 °C) temperatures (Fig. 2a and d), the acoustics revealed a distinct epipelagic layer in 20–40 m depth at the Gakkel Ridge (Fig. 4c, d), possibly consisting of organisms associated with a bloom situation. Ice algae blooms at the ice-water interface are common^62^, but also under-ice phytoplankton blooms occur at low magnitudes in this area and have been found to coincide with a shallowing of the pycnocline to 50 m^63^. Pelagic algal blooms generally occur after ice breakup, which often is as late as August- early September in these northern regions^64^. The trawls hauls do not overlap with the most intense backscattering in the epipelagic layer, and hence, cannot directly corroborate the acoustic data.

A mesopelagic scattering layer was also evident in these regions, although very weak (Fig. 4c, d). The fine meshed macroplankton trawl showed copepods and euphausiids, together with some cnidarians at ca. 350 m depth and, to our knowledge, the northernmost record of *Nematoscelis megalops* (Table 2). However, both the macroplankton and the Harstad trawl catches revealed epi- and mesofauna dominated by arctic species (Table S2 and Fig. 5), which can possibly be related to the Transpolar Drift^27^ (Fig. 1b). Earlier studies have hypothesised that both arctic amphipods (albeit the more sympagic species)^65^ and polar cod^9^ follow the ice drift from the east toward the western Nansen Basin.

### Amundsen Basin

Both trawl catches (Fig. 3) and acoustic registrations (Fig. 4e, f) revealed very low concentrations of zooplankton and fish when entering the extensively ice covered (> 90%), cold and highly stratified (Fig. 2d, e) southern Amundsen Basin (AB1). No catches were taken to corroborate the acoustic peak observed in 25–30 m depth in the southern Amundsen Basin (Fig. 4e), but the macroplankton trawl from the deeper layers revealed a high biomass of copepods in this region (Table S2).

The catch in the mesopelagic layer in the Amundsen Basin consisted of a mixture of gelatinous and crustacean plankton and chaetognaths, and the only fish caught were a few individuals of glacier lanternfish in the southern parts of this Basin (Figs. 3 and 5). However, the observed TS_38_ peak at – 55 to 54 dB (Fig. S17) were considerably stronger than the previous estimates of about − 62 dB for adult glacier lanternfish^66^. Since the TS data were filtered to be within 2° of the acoustic axis before analysis, this result should not be biased due to reduced signal-to-noise ratio in the outer parts of the beam. Furthermore, similar (− 55 to 52 dB) modes were evident at all locations south of AB1. If these stronger echoes came from for instance polar cod, our TS_38_ data imply that polar cod of lengths 6–9 cm^51^ were present at most locations, although in too low concentrations to be caught by the trawls.

Subarctic-boreal species such as the boreal Atlantic expatriate^11^ euphausiid *Meganyctiphanes norvegica* were observed all the way to 87.5^o ^N in the Amundsen Basin (Table 2). These findings add support to our hypothesis that the pelagic composition is driven by dominating water mass origins, but with presence of subarctic-boreal species also in the northernmost parts. Moreover, since the strong topographic steering cause the Atlantic Water to mainly follow slopes and ridges rather than crossing the deep basins^26,28^ (Fig. 1a, c), it is more likely that these subarctic-boreal species reached the northernmost regions by advection through the eastern Nansen and possibly Amundsen Basins. The long advection times from Fram Strait (2–5 years) and the Barents Sea (10–15 years) to the Amundsen Basin^67^ can imply that the krill species currently can live their full life cycle in the Arctic. Nevertheless, as *M. norvegica* has a 3+ years life cycle^68^, firm conclusions on this cannot be made. Increased capacity to complete the life-cycle in the Arctic has recently been proposed for another boreal-Atlantic zooplankton species, *Calanus finmarchicus*^69^.

In the central Amundsen Basin (AB2) the catches revealed a strong presence of gelatinous plankton (ctenophores), again including subarctic-boreal species (Fig. 5). Overwintering individuals of three ctenophore species have been observed in the Chukchi Sea, leading to a proposed hypothesis that they can survive the winter under sea ice in the coastal Arctic by the continued availability of prey related to high productivity, including production by ice algae^70^. Ctenophores had in general a wide distribution in the Nansen and Amundsen basins and were observed at all locations (Fig. 5, Table 2). These observations are consistent with those from the upper 100 m of the Canada Basin^71^. The finer meshed macroplankton trawl also showed presence of *Calanus* spp., *Themisto* spp., *Pseudosagitta* and *Eukrohnia* spp. (Table S2). These comparatively small organisms are clearly poorly represented in the Harstad trawl which is designed to capture fish.

### Regional differences within the European Arctic

Our results show that the densities of both fish and their prey organisms in the Nansen and Amundsen Basins are very low compared to regions further south. The observed catch rates are two orders of magnitude lower than in the slope current north of Svalbard^19,21^. Also the acoustic backscattering is two orders of magnitude less than in northern Svalbard waters^22,58,72^, a region which in turn has backscattering an order of magnitude less than the Norwegian Sea^21^. This confirms our hypothesis that pelagic fishes and large zooplankton in the CAO have lower abundance compared to the south, although the abundances are even lower than we expected. Moreover, recent studies have shown lower densities in the western Nansen and Amundsen Basins as compared to their eastern parts^13^. Thus, despite being closer to the inflow region in Fram Strait, the density of fish and other pelagic organisms in the deep western Nansen Basin seem to be lower than further east. At the same time, the biogeographic composition reflects connectivity to the Atlantic inflow. It remains unclear if the addition of species not found earlier in the study region is a result of strengthened or more frequent inflow or increased research effort.

Substantial warming, subsequent sea ice loss and increased influence of subarctic-boreal species have been documented in the western Nansen Basin slope region^2,34,53^. However, for the off-slope deep Nansen Basin, the largest changes in summer sea ice have occurred in the eastern parts^1^. Changes in summer sea ice in the deep western Nansen Basin are much smaller due to the Transpolar Drift continuously bringing sea ice westwards into the region and further towards Fram Strait^27^ (Fig. 1b). Moreover, the eastern Nansen and Amundsen Basins are also influenced by the Atlantic inflow from the Barents Sea (Fig. 1a). Both the Barents Sea and the eastern Nansen Basin are currently experiencing a pronounced Atlantification with an associated borealization of the ecosystem^2,73^. Therefore, the eastern parts of these basins may be more productive and have better living conditions for subarctic-boreal species than their western parts.

### Sampling in ice covered waters

Acoustic monitoring and net sampling in ice-covered waters pose challenges. Utilizing multi-frequency acoustic data from research vessels is a major step forward methodologically that will facilitate mapping and monitoring of biota in ice-covered waters. However, the high noise and blocking of the acoustic signal generated by the ice-breaking process cause limitations. When lying still, however, the instrumentation provided acoustic data with a signal to noise ratio acceptable for use at several frequencies in the upper water column, and with the lowest frequencies (18 and 38 kHz) down to at least 500 m. The same conclusion was drawn from using 18 kHz acoustic data from a CAO crossing with R/V Oden^23^. We note, however, that because of the reduction in signal to noise ratio with range, only the TS of larger organisms were detected at a few hundred meters.

Pelagic trawling in ice-covered waters with ordinary equipment is challenging because ice floes floating behind the vessel can easily destroy the net during deployment or retrieval. Under-ice trawl samples have previously been obtained with a Surface and Under Ice Trawl^9,74^, but pelagic fish trawls have so far not been used in the CAO due to its thick ice cover^23^. Our results show that only modest adaptations of an ordinary pelagic trawl (Harstad trawl) used routinely on Norwegian research vessels for abundance and biomass estimations of fish, functioned well in ice-covered waters. We used ice trawl gallows to move the tow blocks into the centre of the stern of the vessel, thereby closing the trawl before it reached the surface during retrieval and before sinking beneath the surface during setting. In addition, the trawl was slightly modified by reducing the flotation and adding weight to the cod-end. This made the trawl hang almost vertically from the stern of the vessel during deployment and retrieval, which further reduced the problem with sea ice entering the trawl. For the smaller macroplankton trawl, the only modification made was adding 40 kg to the cod-end.

Horizontal towing of a trawl has clear advantages compared to other sampling methods (like gillnets, fishing lines, vertical towed nets or video), particularly for extremely low densities as in the CAO. Towing one to two nautical miles, as during this survey, the Harstad trawl filtered approximately 500.000–1.000.000 m^3^ of water. Nevertheless, with its vertical opening of 10–12 m (Table 1), and relatively short towing times, the trawl is still too small to obtain large catches at these densities, despite using the same towing speed as in ice-free waters. With maximum catch rates of about 325 and 696 g nm^−1^ (Harstad trawl and macroplankton trawl, respectively), there are uncertainties related to whether the catch composition reflects the actual composition of organisms in the sea. The different catchabilities of the Harstad and macroplankton trawls add further complexity to this issue. Using a larger trawl would undoubtedly be a more efficient approach for catching fish but would also result in severe complications regarding operations in ice-covered waters. For catching smaller organisms (e.g., copepods), smaller nets like WP2 or Multinet with 180 µm mesh are more efficient.

### Baseline mapping of an ice-covered ecosystem

In 2021, a CAO Fisheries Agreement entered into force (www.fao.org/faolex/results/details/en/c/LEX-FAOC199323). In addition to preventing fisheries in the high seas of the CAO at least up to 2037, the agreement calls for the establishment of a Joint Program of Scientific Research and Monitoring with the aim of improving the understanding of the ecosystems. A draft plan for such a program emphasised the need for baseline mapping, monitoring and research^8^.

Using trawls and rigging modified to use in ice covered waters, in combination with state-of-the art multi-frequency acoustics, our results directly contribute to the baseline mapping of the CAO ecosystem while it is even today still ice-covered during most of the year. Our results show that, at present, the densities of both ecologically and economically important fish and their prey organisms in the western Nansen and Amundsen Basins of the CAO are extremely low. Model studies also conclude that the production potential of the region is too low to warrant commercial fisheries in the future^2,7^.

## Material and methods

### Trawl specifications

Two pelagic trawls were used and modified to reduce damage from the ice at the surface. The standard Norwegian pelagic trawl for pelagic fish (capelin, herring and juveniles) in northern areas (Harstad 320 trawl^75^) was used with modified rigging. The trawl (~ 250 m^2^ opening area) is graded from 200 mm mesh in the front of the trawl to 60 mm before the cod-end. The cod-end has an 8-mm mesh net. The modified rigging included wire instead of spectra sweeps (both 60 m long), reducing the total buoyancy of the trawl net (from 1047 to 50 kg), and applying 150 kg weights in front of each lower wing and a 40 kg weight at the end of the cod-end. The ice-modified Harstad trawl had an average opening height of between 9.6 and 12.4 m and an average door spread of 44.7–71.5 m (Table 1).

The macroplankton 92 m trawl (trawl opening area of ~ 36 m^2^; refs.^76,77^ was used with ordinary rigging, except that 40 kg weight was added at the back part of the cod-end. The macroplankton trawl is a non-graded trawl with 8 mm mesh opening (EN ISO 1107:2003) from the front of the trawl to the cod-end.

### Trawling operations

Trawling was conducted in leads with very thin new ice (Supplementary section 2). Fishing depth was determined by acoustic registrations on the EK80. With weak acoustic registrations, one trawl was set at ~ 50 m, and another one in the mesopelagic depth layer of 300–450 m^23^. Prior to each deployment, the upper wings of the trawl were mounted together with thin rope to prevent ice from entering the trawl during shooting. This rope broke when the trawl doors were deployed due to the horizontal spread forces. Vessel speed was reduced during shooting of the trawl and the door release to increase the descent rate of the trawl (i.e., the trawl was hanging nearly vertically behind the vessel). When the doors were at approximately 4–6 m depth, the vessel increased its speed and the warps were deployed as during ordinary pelagic trawling. The Harstad trawl was towed at 1.5 ms^−1^ while the macroplankton trawl was towed at 1.0 ms^−1^ (speed over ground—SOG) for ~ 30 min at different depths (Table 1), which is the same speeds as used in ice-free waters. As the trawl gear approached the surface during haul back, the vessel reduced its speed to allow the doors and trawl to come on deck at a vertical angle close to the stern of the vessel (i.e., the trawl was hanging nearly vertically behind the vessel). The vessel used Seaonics ice trawl gallows (automated system to move the tow blocks into the centre of the stern of the vessel) when the sea ice thickness and volume was high. The vertical opening of the trawl net (Harstad trawl only), trawl depth, door spread and door depth were measured with acoustic trawl instrumentation (SCANMAR AS, Åsgårdstand, Norway).

### Biological data

Trawl catches were sorted immediately, and organisms were identified to species level when possible. Fish were weighed and length measured. Excess water was gently filtered from the macrozooplankton (euphausiids, amphipods, etc.) samples using a 1 mm sieve. Representative subsamples of 100 g were preserved in 4% hexamine-buffered formalin solution in 500 ml plastic bottles. The preserved samples were species identified length measured, when possible, at the IMR laboratory. Institute of Marine Research fully adheres to Norwegian laws relevant to Ethics in Science as well as Animal Welfare. Institute of Marine Research is a governmental research institute with given permission to perform research cruises including fish samplings by the Norwegian Government. All methods were carried out in accordance with relevant guidelines and regulations. All methods are reported in accordance with ARRIVE guidelines. No experiments were carried out on live organisms.

The sampling protocols were approved by Institute of Marine Research (IMR Quality system https://kvalitet.hi.no/docs/pub/DOK06839.pdf) and UiT The Arctic University of Norway leading the Nansen Legacy project (https://doi.org/10.7557/nlrs.5882).

### Acoustical data

Acoustical data were obtained from a Simrad EK80 echosounder mounted behind an ice window under the hull. The echosounder was calibrated according to standard procedures^78^ on 19.01.2021. Only data from periods when the ship was stationary were used due to mechanical noise when the vessel was moving through sea ice. An hour of acoustic data without noise was selected from as near the trawl locations as possible (Fig. 1c, Table S1). Applying a threshold of − 90 dB gave echosounder data down to at least 500 m for the frequencies 18 and 38 kHz. The usable range was less for the higher frequencies (for 70 kHz about 400 m, for 120 kHz about 200 m, for 200 kHz about 100 m, and for 333 kHz less than 50 m).

The echo integrator threshold in terms of S_v_ in dB was set at − 90 dB re 1 m^−1^ (ref.^50^). The 38 kHz frequency was used to determine backscatter intensity. Analysis was done in the postprocessing tool LSSS v. 2.11.0 (ref.^79^). The backscattering data output were in the form of s_A_, and all definitions and notations of the acoustic quantities were according to Maclennan^50^ (see Supplementary section 3 for more details). The data were integrated over the time period and stored at a grid of 10 m depth and 1 h (3600 s).

To illustrate how the total acoustic backscatter was distributed along an axis from weak scatterers to strong scatterers, the s_A_ was classified into five classes according to their s_V_ by sequential thresholding at 38 kHz. The five categories used were S70, S75, S80, S85 and S90 (Supplementary section 3), where S70 represent the organisms with strongest target strength (TS) and/or the highest density, while S90 represent the organism with weakest TS and/or the lowest density.

Various types of organisms of the same size often have different acoustic properties at different frequencies, and the distribution of types of scattering organisms was assessed using frequency response in s_V_. The different frequencies were considered down to their acceptable ranges, and we give the relative frequency response between 18 and 38 kHz (rf_18/38_) when feasible.

We identified type of organisms present by measurements of TS of individual scatterers. We mainly considered TS measurement from the 38 kHz, but TS from 18 kHz were also assessed when available. The higher frequencies were only considered when we recorded extraordinary registrations (like at NB1). The TS measurements were identified in LSSS using the following settings: Detector type SED (Single echo detector), Min TS − 70 dB, Pulse length determination Level: 6 dB, Minimum echo length: 0.5 (relative to pulse length), Maximum echo length 2.0 (relative to pulse length), Max gain compensation 6 dB, Phase Deviation Check: On. Max phase deviation: 8 steps. Thereafter, we filtered the data restricting them to be within 2° off axis to avoid TS distributions being biased towards higher values due to reduced signal-to-noise ratio in the outer parts of the beam.

### Environmental data

Both sea ice concentration and drift during the cruise period were obtained from the EUMETSAT Ocean and Sea Ice Satellite Application Facility (OSI SAF), in collaboration with ESA CCI (https://osi-saf.eumetsat.int/products/sea-ice-products). Daily sea ice concentration from passive microwave satellite data is from the interim climate data record (OSI-430-b^80^) and is provided daily on a grid with a resolution of 25 km. Sea ice drift velocities are from the low-resolution sea ice drift product (OSI-405-c) and are daily at a resolution of 62.5 km.

Observations of ocean currents were obtained from the vessel mounted RDI 150 kHz Acoustic Doppler Current Profiler (ADCP) mounted behind the ice window under the hull. Post-processing of ADCP data was performed using the Common Oceanographic Data Access System^81^. Tides were removed using the AOTIM tidal model^82^. The vessel mounted ADCP returned data from 25 to 250–350 m depth. Relatively continuous ADCP data were recorded during transit up to about 82.5^o ^N. To the north of this, VM-ADCP only delivered data when the vessel was stationary. To keep the high resolution when crossing the Atlantic Water slope current, ADCP data from 80.5^o ^N to 82.5^o ^N were gridded to an along-track vector with 10 km horizontal resolution. To the north of this, ADCP data were extracted from each CTD station (within 0.5 km of the station), and then averaged to obtain a vertical profile of velocity at the CTD station. This returned velocity along a vector with 10 km horizontal resolution up to 82.5^o ^N and the resolution of the CTD stations to the north of this.

Temperature and salinity were measured using a Seabird 911plus CTD at and between the trawl stations (Figs. 1c, 4d, e). The CTD was equipped with a WET Labs Fluorometer and a rosette system for collecting water samples. The conductivity, temperature, depth, and oxygen sensors are serviced and calibrated once a year by the manufacturer (Seabird). In situ water samples for salinity calibration (conductivity sensor) were taken at every station at maximum depth.

## Supplementary Information

Supplementary Information 1.

Supplementary Tables.

## Data Availability

The cruise data analysed in the study are available at Norwegian Marine Data Centre. CTD data can be found at https://doi.org/10.21335/NMDC-1814168447, ADCP data at https://doi.org/10.21335/NMDC-1175579976, trawl catch data at https://doi.org/10.21335/NMDC-194651742 and acoustic data at https://doi.org/10.21335/NMDC-1275935147. Sea ice data are available at https://osi-saf.eumetsat.int/products/sea-ice-products.

## References

[1] Stroeve J, Notz D (2018). Changing state of Arctic sea ice across all seasons. Environ. Res. Lett..

[2] Polyakov IV (2020). Borealization of the Arctic Ocean in response to anomalous advection from sub-Arctic seas. Front. Mar. Sci..

[3] Lannuzel D (2020). The future of Arctic sea-ice biogeochemistry and ice-associated ecosystems. Nat. Clim. Change..

[4] Macias-Fauria M, Post E (2018). Effects of sea ice on Arctic biota: An emerging crisis discipline. Biol. Lett..

[5] Kohlbach D (2016). The importance of ice algae-produced carbon in the central Arctic Ocean ecosystem: Food web relationships revealed by lipid and stable isotope analyses. Limnol. Oceanogr..

[6] Søreide JE (2013). Sympagic-pelagic-benthic coupling in Arctic and Atlantic waters around Svalbard revealed by stable isotopic and fatty acid tracers. Mar. Biol. Res..

[7] Slagstad D, Wassmann PFJ, Ellingsen I (2015). Physical constrains and productivity in the future Arctic Ocean. Front. Mar. Sci..

[8] FISCAO. *Final Report of the Fifth Meeting of Scientific Experts on Fish Stocks in the Central Arctic Ocean*. https://apps-afsc.fisheries.noaa.gov/documents/Arctic_fish_stocks_fifth_meeting/508_Documents/508_Final_report_of_the_505th_FiSCAO_meeting.pdf (2018).

[9] David C (2016). Under-ice distribution of polar cod *Boreogadus saida* in the central Arctic Ocean and their association with sea-ice habitat properties. Polar Biol..

[10] Gradinger R (1999). Vertical fine structure of the biomass and composition of algal communities in Arctic pack ice. Mar. Biol..

[11] Kosobokova KN, Hopcroft RR, Hirche H-J (2011). Patterns of zooplankton diversity through the depths of the Arctic’s central basins. Mar Biodivers..

[12] Mumm N (1998). Breaking the ice: Large-scale distribution of mesozooplankton after a decade of Arctic and transpolar cruises. Polar Biol..

[13] Snoeijs-Leijonmalm P (2022). Unexpected fish and squid in the central Arctic deep scattering layer. Sci. Adv..

[14] David C, Lange B, Rabe B, Flores H (2015). Community structure of under-ice fauna in the Eurasian central Arctic Ocean in relation to environmental properties of sea-ice habitats. Mar. Ecol. Prog. Ser..

[15] Gosselin M, Levasseur M, Wheeler PA, Horner RA, Booth BC (1997). New measurements of phytoplankton and ice algal production in the Arctic Ocean. Deep-Sea Res. Part.

[16] Ardyna M, Arrigo KR (2020). Phytoplankton dynamics in a changing Arctic Ocean. Nat. Clim. Change..

[17] Hays, G. C. In *Migrations and Dispersal of Marine Organisms.* (eds Jones, M. B. *et al.*) 163–170 (Springer).

[18] Irigoien X (2014). Large mesopelagic fishes biomass and trophic efficiency in the open ocean. Nat. Commun..

[19] Geoffroy M (2019). Mesopelagic sound scattering layers of the high arctic: Seasonal variations in biomass, species assemblage, and trophic relationships. Front. Mar. Sci..

[20] Gjøsæter H, Wiebe PH, Knutsen T, Ingvaldsen RB (2017). Evidence of Diel vertical migration of mesopelagic sound-scattering organisms in the Arctic. Front. Mar. Sci..

[21] Knutsen T, Wiebe PH, Gjøsæter H, Ingvaldsen RB, Lien G (2017). High latitude epipelagic and mesopelagic scattering layers—a reference for future arctic ecosystem change. Front. Mar. Sci..

[22] Priou P (2021). Dense mesopelagic sound scattering layer and vertical segregation of pelagic organisms at the Arctic-Atlantic gateway during the midnight sun. Prog. Oceanogr..

[23] Snoeijs-Leijonmalm P (2021). A deep scattering layer under the North Pole pack ice. Prog. Oceanogr..

[24] St-John MA (2016). A dark hole in our understanding of marine ecosystems and their services: Perspectives from the mesopelagic community. Front. Mar. Sci..

[25] Fransson, A. *et al.**Joint cruise 2-2 2021: Cruise report. The Nansen Legacy Report Series, 30/2022*. 10.7557/nlrs.6413 (2022).

[26] Rudels B (2013). Observations of water masses and circulation with focus on the Eurasian Basin of the Arctic Ocean from the 1990s to the late 2000s. Ocean Sci..

[27] Krumpen T (2019). Arctic warming interrupts the Transpolar Drift and affects long-range transport of sea ice and ice-rafted matter. Sci. Rep..

[28] Aagaard K (1989). A synthesis of the Arctic Ocean circulation. Rapp. P.-V. Rcun. Cons. int. Explor. Mer..

[29] Perez-Hernandez MD (2017). The Atlantic Water boundary current north of Svalbard in late summer. J. Geophys. Res..

[30] Våge K (2016). The Atlantic Water boundary current in the Nansen Basin: Transport and mechanisms of lateral exchange. J. Geophys. Res..

[31] Crews L, Sundfjord A, Albretsen J, Hattermann T (2018). Mesoscale Eddy Activity and Transport in the Atlantic Water Inflow Region North of Svalbard. J. Geophys. Res..

[32] Kolås EH, Koenig Z, Fer I, Nilsen F, Marnela M (2020). Structure and Transport of Atlantic Water North of Svalbard From Observations in Summer and Fall 2018. J. Geophys. Res..

[33] Basedow SL (2018). Seasonal variation in transport of zooplankton into the arctic basin through the atlantic gateway. Fram Strait. Front. Mar. Sci..

[34] Vernet M, Carstensen J, Reigstad M, Svensen C (2020). Editorial: Carbon bridge to the Arctic. Front. Mar. Sci..

[35] Wassmann P (2015). The contiguous domains of Arctic Ocean advection: Trails of life and death. Prog. Oceanogr..

[36] Wassmann P, Slagstad D, Ellingsen I (2019). Advection of mesozooplankton into the northern svalbard shelf region. Front. Mar. Sci..

[37] Auel H (2004). Egg size and reproductive adaptations among Arctic deep-sea copepods (Calanoida, *Paraeuchaeta*). Helgol. Mar. Res..

[38] Gluchowska M (2016). Zooplankton in Svalbard fjords on the Atlantic-Arctic boundary. Polar Biol..

[39] Wang Y-G, Tseng L-C, Lin M, Hwang J-S (2019). Vertical and geographic distribution of copepod communities at late summer in the Amerasian Basin. Arctic Ocean. Plos One..

[40] Gislason A, Silva T (2012). Abundance, composition, and development of zooplankton in the Subarctic Iceland Sea in 2006, 2007, and 2008. ICES J. Mar. Sci..

[41] Zhukova NG, Nesterova VN, Prokopchuk IP, Rudneva GB (2009). Winter distribution of euphausiids (Euphausiacea) in the Barents Sea (2000–2005). Deep-Sea Res. Part.

[42] Dalpadado P, Skjoldal HR (1996). Abundance, maturity and growth of the krill species *Thysanoessa inermis* and *T. longicaudata* in the Barents Sea. Mar. Ecol. Prog. Ser..

[43] Koszteyn J, Timofeev S, Węsławski JM, Malinga B (1995). Size structure of *Themisto abyssorum* Boeck and *Themisto libellula* (Mandt) populations in European Arctic seas. Polar Biol..

[44] Dalpadado P, Borkner N, Bogstad B, Mehl S (2001). Distribution of Themisto (Amphipoda) spp. in the Barents Sea and predator-prey interactions. ICES J. Mar. Sci..

[45] Macnaughton MO, Thormar J, Berge J (2007). Sympagic amphipods in the Arctic pack ice: Redescriptions of *Eusirus holmii* Hansen, 1887 and *Pleusymtes karstensi* (Barnard, 1959). Polar Biol..

[46] Kraft A, Graeve M, Janssen D, Greenacre M, Falk-Petersen S (2015). Arctic pelagic amphipods: Lipid dynamics and life strategy. J. Plankton Res..

[47] Kreibich T, Hagen W, Saborowski R (2010). Food utilization of two pelagic crustaceans in the Greenland Sea: *Meganyctiphanes norvegica* (Euphausiacea) and *Hymenodora glacialis* (Decapoda, Caridea). Mar. Ecol. Prog. Ser..

[48] Geoffroy M (2018). Increased occurrence of the jellyfish *Periphylla periphylla* in the European high Arctic. Polar Biol..

[49] Grigor JJ, Søreide JE, Varpe Ø (2014). Seasonal ecology and life-history strategy of the high-latitude predatory zooplankter *Parasagitta elegans*. Mar. Ecol. Prog. Ser..

[50] Maclennan DN, Fernandes PG, Dalen J (2002). A consistent approach to definitions and symbols in fisheries acoustics. ICES J. Mar. Sci..

[51] Gjøsæter, H. & Ushakov, N. G. *Acoustic estimates of the Barents Sea Arctic cod Stock (Boreogadus saida). Forage Fishes in Marine Ecosystems. Alaska Sea Grant Collage Program, University of Alaska Fairbanks* 97:01, 485–504 (1997).

[52] Raskoff KA, Hopcroft RR, Kosobokova KN, Purcell JE, Youngbluth M (2010). Jellies under ice: ROV observations from the Arctic 2005 hidden ocean expedition. Deep-Sea Res. Part.

[53] Bluhm BA (2020). The Pan-Arctic continental slope: Sharp gradients of physical processes affect pelagic and benthic ecosystems. Front. Mar. Sci..

[54] Hop H (2019). Pelagic ecosystem characteristics across the atlantic water boundary current from Rijpfjorden, Svalbard, to the Arctic Ocean During Summer (2010–2014). Front. Mar. Sci..

[55] Mumm N (1993). Composition and distribution of mesozooplankton in the Nansen Basin, Arctic Ocean, during summer. Polar Biol..

[56] Ona E, Nielsen J (2022). Acoustic detection of the Greenland shark (*Somniosus microcephalus*) using multifrequency split beam echosounder in Svalbard waters. Prog. Oceanogr..

[57] Gjøsæter H, Ingvaldsen R, Christiansen JS (2020). Acoustic scattering layers reveal a faunal connection across the Fram Strait. Prog. Oceanogr..

[58] Ingvaldsen RB, Gjosaeter H, Ona E, Michalsen K (2017). Atlantic cod (*Gadus morhua*) feeding over deep water in the high Arctic. Polar Biol..

[59] Chawarski J, Klevjer TA, Coté D, Geoffroy M (2022). Evidence of temperature control on mesopelagic fish and zooplankton communities at high latitudes. Front. Mar. Sci..

[60] Chernova NV (2017). Catching of Greenland halibut *Reinhardtius hippoglossoides* (Pleuronectidae) on the shelf edge of the Laptev and East Siberian Seas. J. Ichthyol..

[61] Benzik AN, Budanova LK, Orlov AM (2022). Hard life in cold waters: Size distribution and gonads show that Greenland halibut temporarily inhabit the Siberian Arctic. Water Biol. Secur..

[62] Olsen LM (2019). A red tide in the pack ice of the Arctic Ocean. Sci. Rep..

[63] Assmy P (2017). Leads in Arctic pack ice enable early phytoplankton blooms below snow-covered sea ice. Sci. Rep..

[64] Leu E, Søreide JE, Hessen DO, Falk-Petersen S, Berge J (2011). Consequences of changing sea-ice cover for primary and secondary producers in the European Arctic shelf seas: Timing, quantity, and quality. Prog. Oceanogr..

[65] Drivdal M (2021). Connections to the deep: Deep vertical migrations, an important part of the life cycle of *Apherusa glacialis*, an arctic ice-associated amphipod. Front. Mar. Sci..

[66] Scoulding B, Chu D, Ona E, Fernandes PG (2015). Target strengths of two abundant mesopelagic fish species. J. Acoust. Soc. Am..

[67] Popova EE, Yool A, Aksenov Y, Coward AC (2013). Role of advection in Arctic Ocean lower trophic dynamics: A modeling perspective. J. Geophys. Res..

[68] Saunders RA, Ingvarsdóttir A, Rasmussen J, Hay SJ, Brierley AS (2007). Regional variation in distribution pattern, population structure and growth rates of *Meganyctiphanes norvegica* and *Thysanoessa longicaudata* in the Irminger Sea, North Atlantic. Prog. Oceanogr..

[69] Tarling GA (2022). Can a key boreal *Calanus* copepod species now complete its life-cycle in the Arctic? Evidence and implications for Arctic food-webs. Ambio.

[70] Purcell JE, Juhl AR, Manko MK, Aumack CF (2018). Overwintering of gelatinous zooplankton in the coastal Arctic Ocean. Mar. Ecol. Prog. Ser..

[71] Purcell JE, Hopcroft RR, Kosobokova KN, Whitledge TE (2010). Distribution, abundance, and predation effects of epipelagic ctenophores and jellyfish in the western Arctic Ocean. Deep-Sea Res. Part.

[72] Solvang HK (2021). Distribution of rorquals and Atlantic cod in relation to their prey in the Norwegian high Arctic. Polar Biol..

[73] Ingvaldsen RB (2021). Physical manifestations and ecological implications of Arctic Atlantification. Nat. Rev. Earth Environ..

[74] Flores H (2011). Macrofauna under sea ice and in the open surface layer of the Lazarev Sea, Southern Ocean. Deep-Sea Res. Part.

[75] Godø OR, Valdemarsen JW, Engås A (1993). Comparison of efficiency of standard and experimental juvenile gadoid sampling trawls. ICES Mar. Sci. Symp..

[76] Klevjer T (2020). Micronekton biomass distribution, improved estimates across four north Atlantic basins. Deep-Sea Res. Part II..

[77] Krafft BA (2010). Distribution and demography of Antarctic krill in the Southeast Atlantic sector of the Southern Ocean during the austral summer 2008. Polar Biol..

[78] Foote KG (1983). Maintaining precision calibrations with optimal copper spheres. J. Acoust. Soc. Am..

[79] Korneliussen RJ (2016). Acoustic identification of marine species using a feature library. Methods Oceanogr..

[80] Lavergne T (2019). Version 2 of the EUMETSAT OSI SAF and ESA CCI sea-ice concentration climate data records. Cryosphere..

[81] Firing, E., Ramada, J. & Caldwell, P. *Processing ADCP Data with the CODAS Software System Version 3.1. Joint Institute for Marine and Atmospheric Research University of Hawaii*. http://currents.soest.hawaii.edu/docs/adcp_doc/index.html (1995).

[82] Padman L, Erofeeva S (2004). A barotropic inverse tidal model for the Arctic Ocean. Geophys. Res. Lett..

