# Supplementary material for: Under-ice observations by trawls and multi-frequency acoustics in the Central Arctic Ocean reveals abundance and composition of pelagic fauna
Source: Sci Rep. 2023 Jan 18;13:1000. doi: 10.1038/s41598-023-27957-x (PMC9849409; doi:10.1038/s41598-023-27957-x)
Supplement: Supplementary file 1 — Supplementary Information 1. [file 41598_2023_27957_MOESM1_ESM.pdf]

Supplementary materials

Under-ice observation by trawls and multi-frequency acoustics in the Central Arctic Ocean reveals abundance and composition of pelagic fauna

Randi B. Ingvaldsen[1], Elena Eriksen[1], Harald Gjøsæter[1], Arill Engås[1], Birte Katarina Schuppe[2], Karen M. Assmann[2], Heather Cannaby[1], Padmini Dalpadado[1], Bodil A. Bluhm[3]

[1]Institute of Marine Research, B.O. Box 1870 Nordnes, 5817 Bergen, Norway
[2]Institute of Marine Research, Framsenteret, Postboks 6606 Stakkevollan, 9296 Tromsø, Norway
[3]UIT The Arctic University of Norway, Tromsø

Contents

Supplementary section 1: Oceanography 2

Supplementary section 2: Trawling 3

Supplementary section 3: Acoustics 15

Methods and analysis 15

Nansen Basin 16

Gakkel Ridge 21

Amundsen Basin 23

References 24

Supplementary section 1: Oceanography

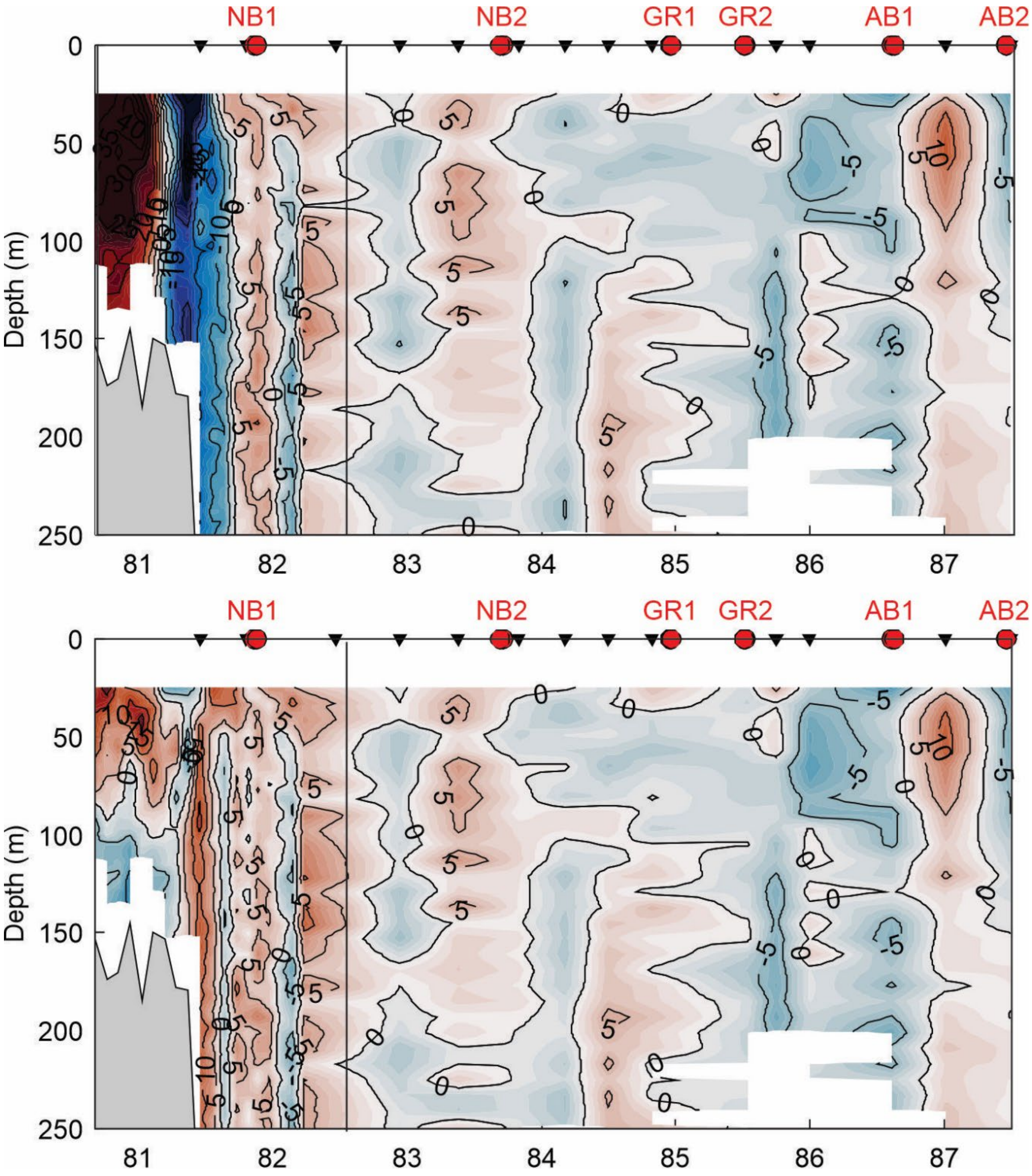

Figure S1. Vessel-mounted ADCP data (in cm/s) showing east-west flow (upper panel, positive towards east) and north-south flow (lower panel, positive towards north). The black line at 82.5°N shows where the horizontal resolution of the ADCP data changes; to the south of this line the horizontal resolution is 10 km while to the north of it the resolution is given by the separation of the CTD stations (shown by ▼). ● show the location of the trawls.

Supplementary section 2: Trawling

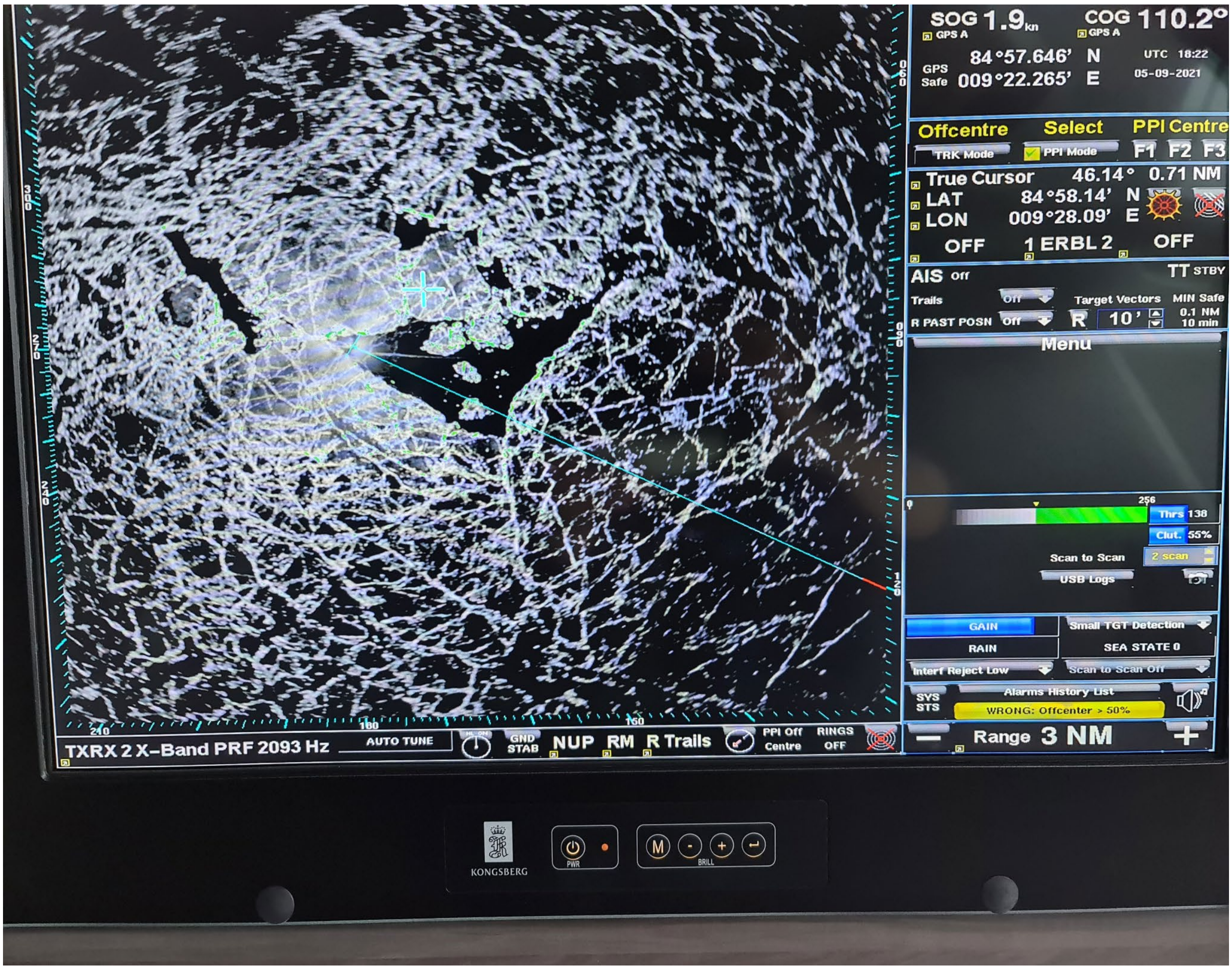

Figure S2. Trawling was conducted in leads with very thin new ice and the picture shows one example of such a lead.

Trawl catches were sorted immediately, and organisms were identified to species level (when possible). In the following pages we document the trawl catches by pictures taken on the vessel. Pictures of *Calanus hyperboreus* and *Paraeuchaeta glacialis* taken in the laboratory are shown in Fig. S15.

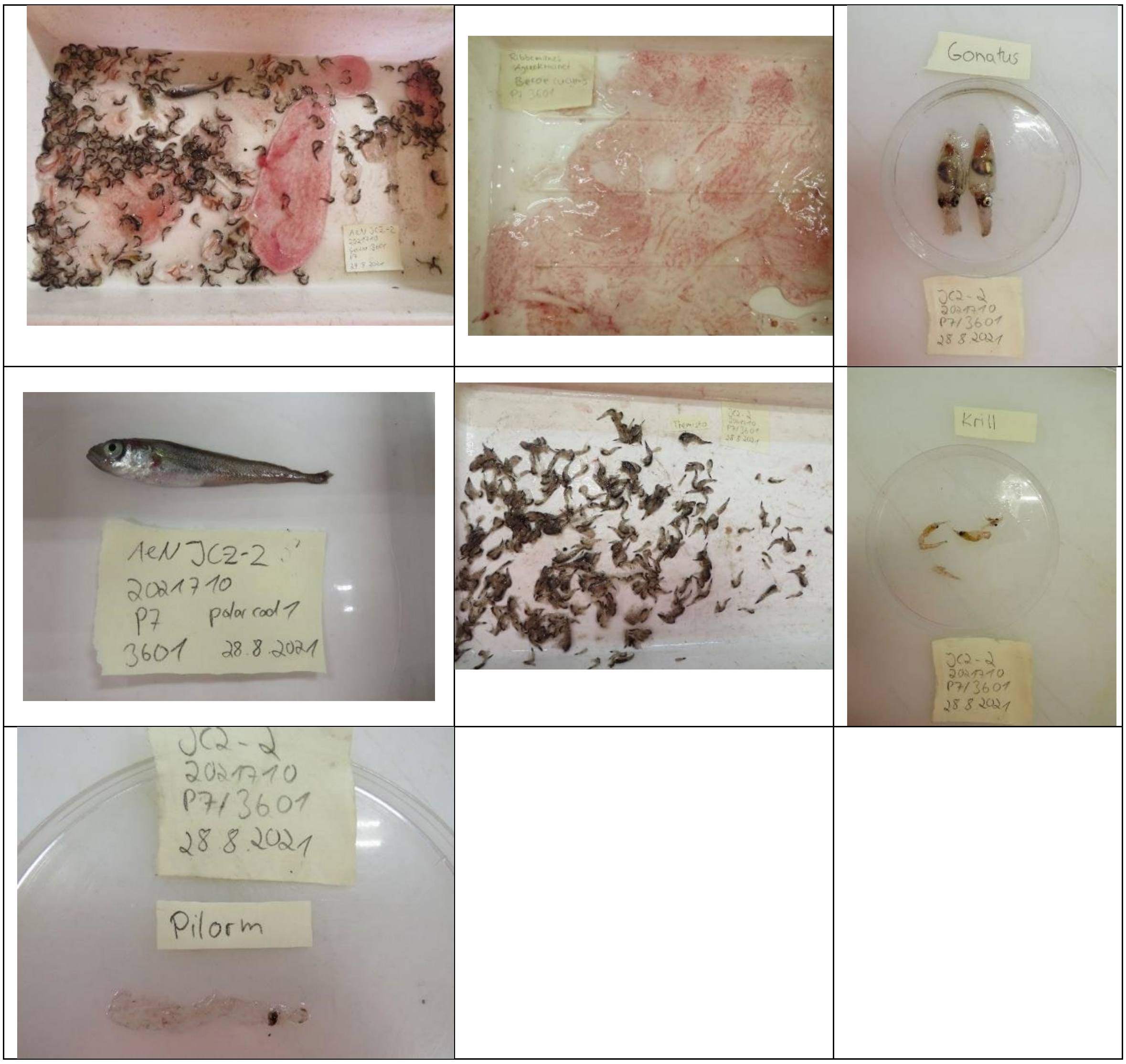

Figure S3. Photos of the catch from NB1e (P7b) 3601 (Harstad trawl with fishing depth 60-40 m).

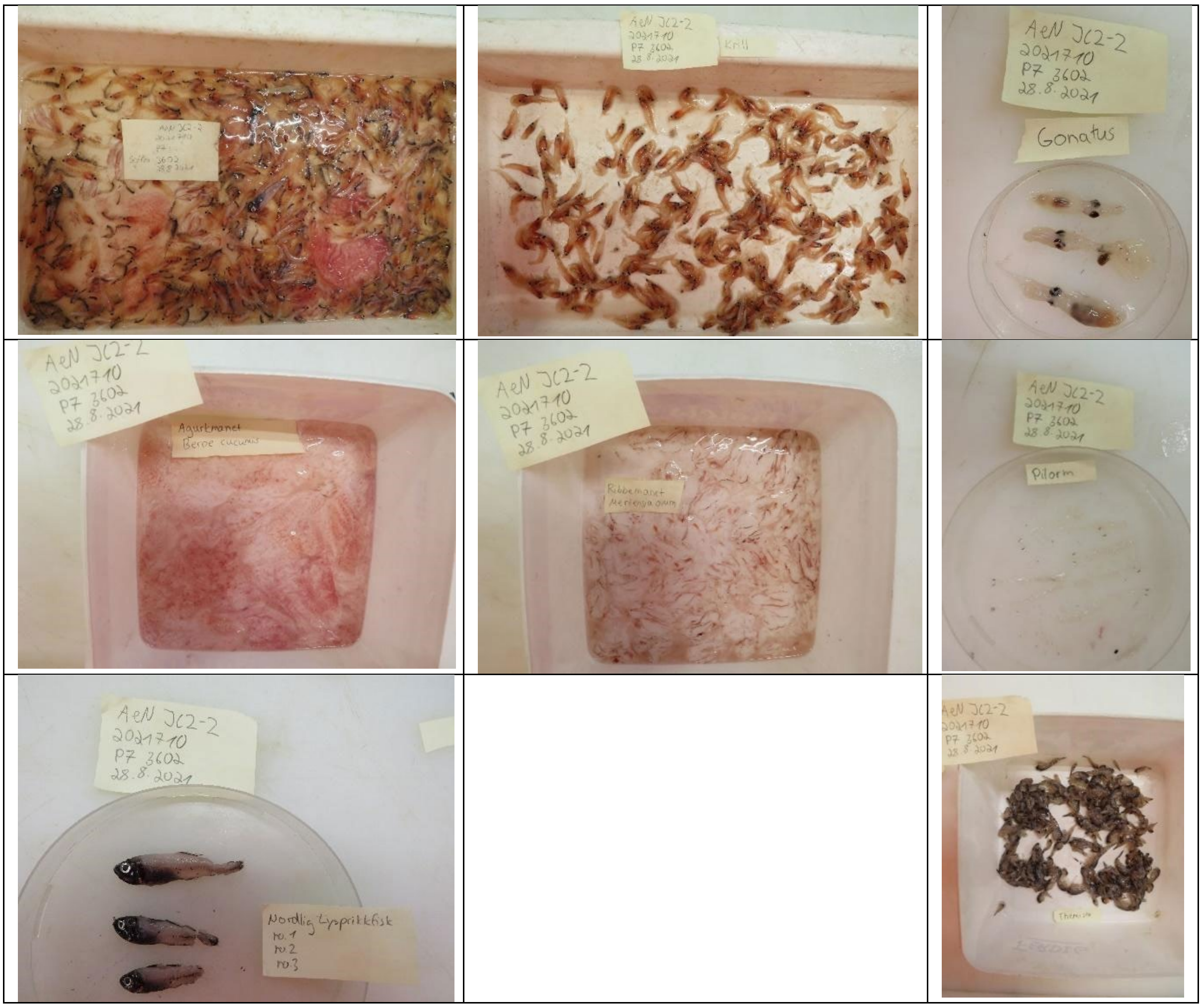

Figure S4. Photos of the catch from NB1m (P7b) 3602 (Harstad trawl with fishing depth 460-450 m).

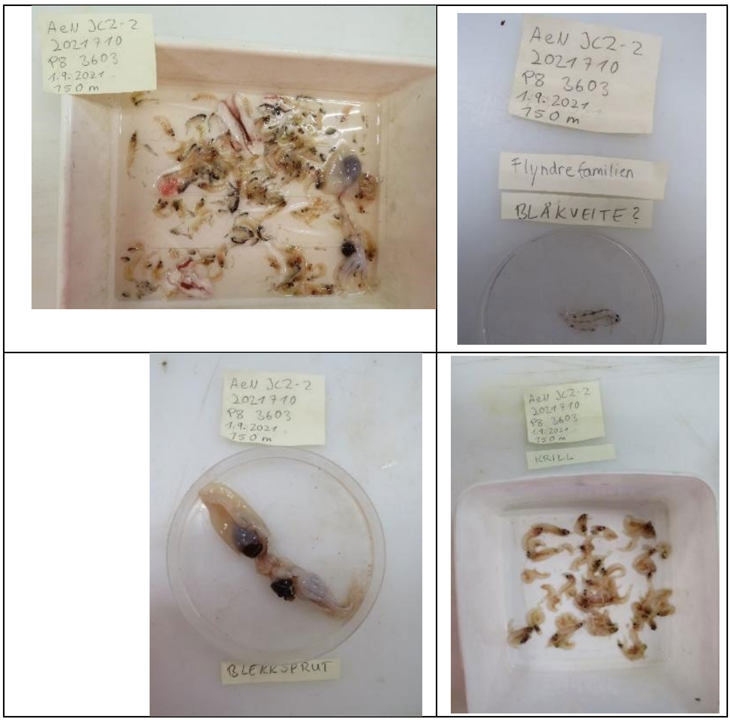

Figure S5. Photos of the catch from NB2e (P8a) 3603 (Harstad trawl with fishing depth 160-150 m).

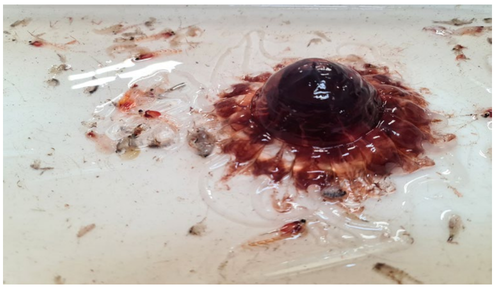

Figure S6. Photo of the catch from NB2m (P8a) 3604 (Harstad trawl with fishing depth 600-450 m).

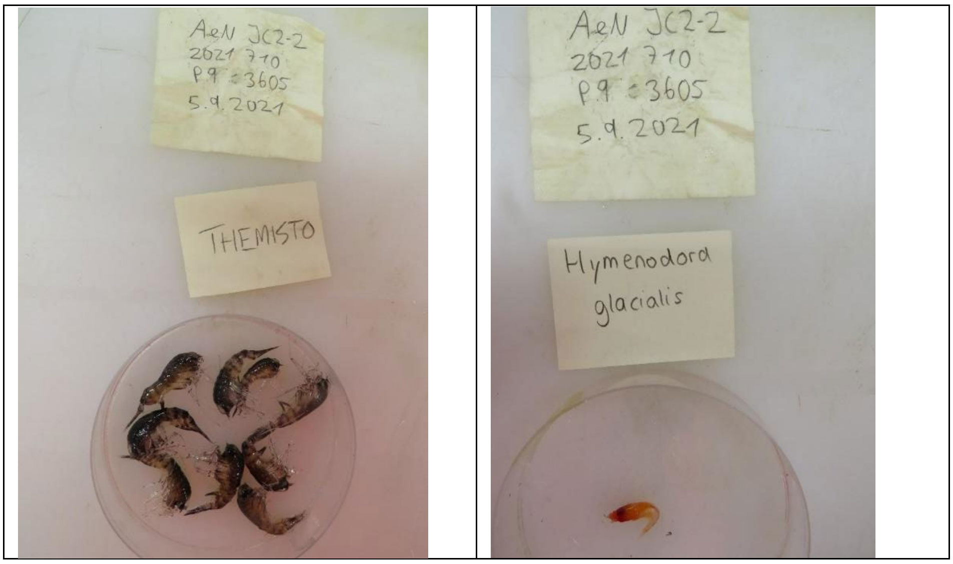

Figure S7. Photos of the catch from GR1m (P9a) 3605 (Harstad trawl with fishing depth 550-400 m).

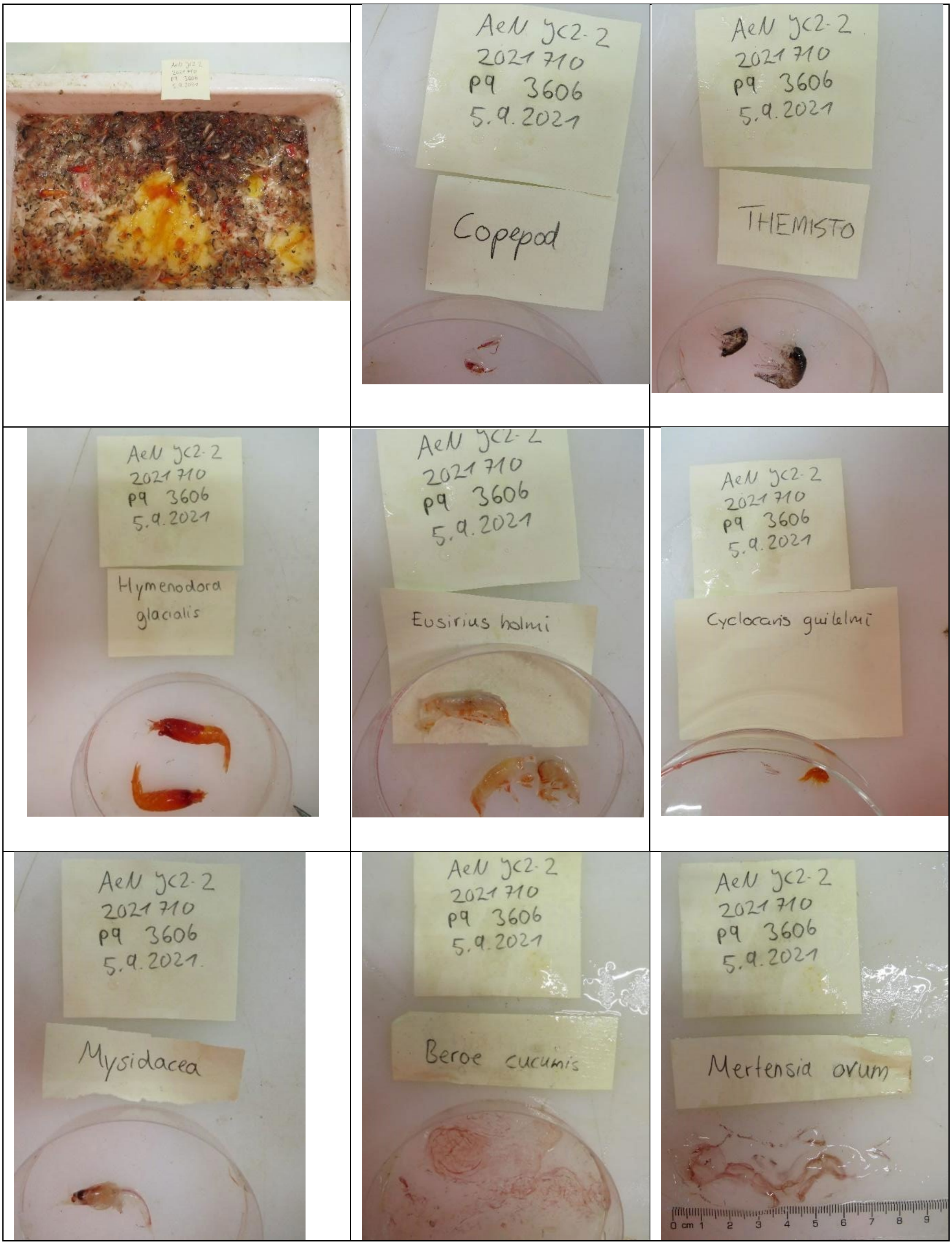

Figure S8. Photos of the catch from GR1mMT (P9a) 3606 (macroplankton trawl with fishing depth 537-390 m).

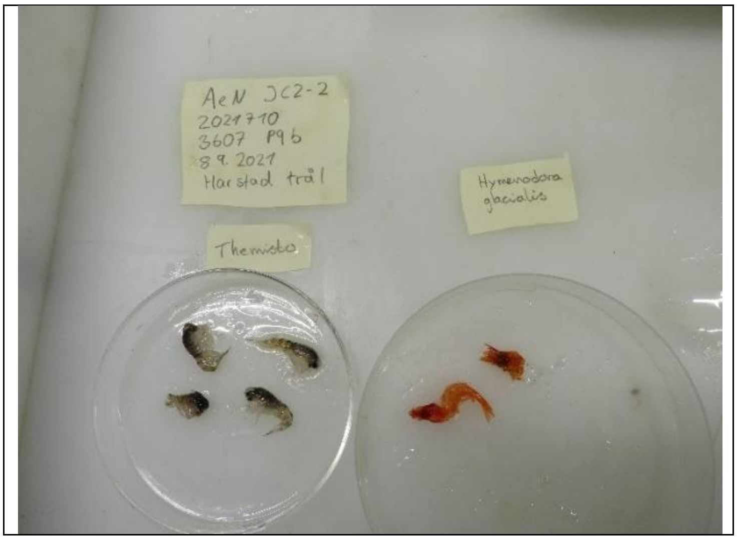

Figure S9. Photo of the catch from GR2e (P9) 3607 (Harstad trawl with fishing depth 211-50 m).

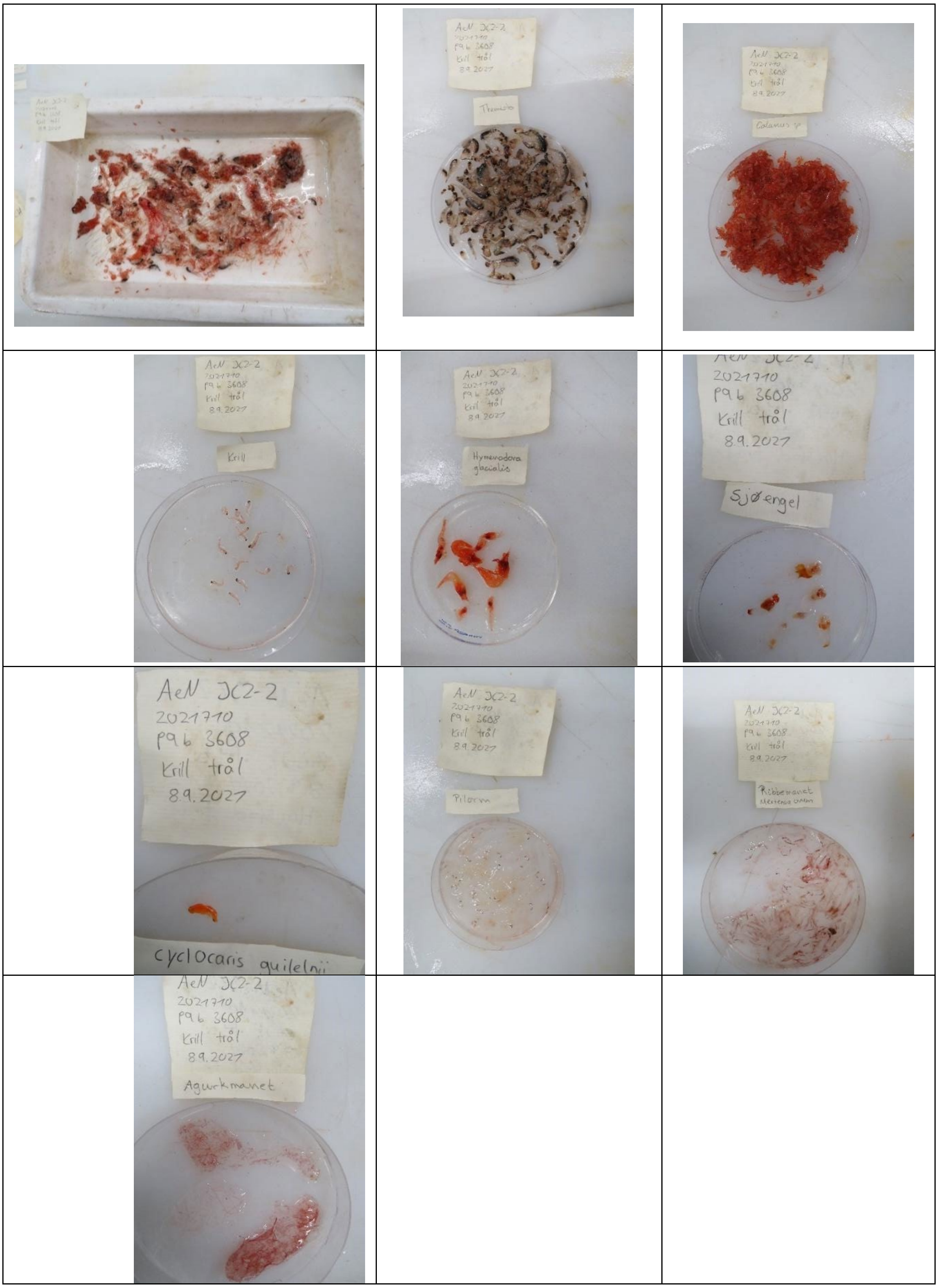

Figure S10. Photos of the catch from GR2emMT (P9) 3608 (macroplankton trawl with fishing depth 347-60 m).

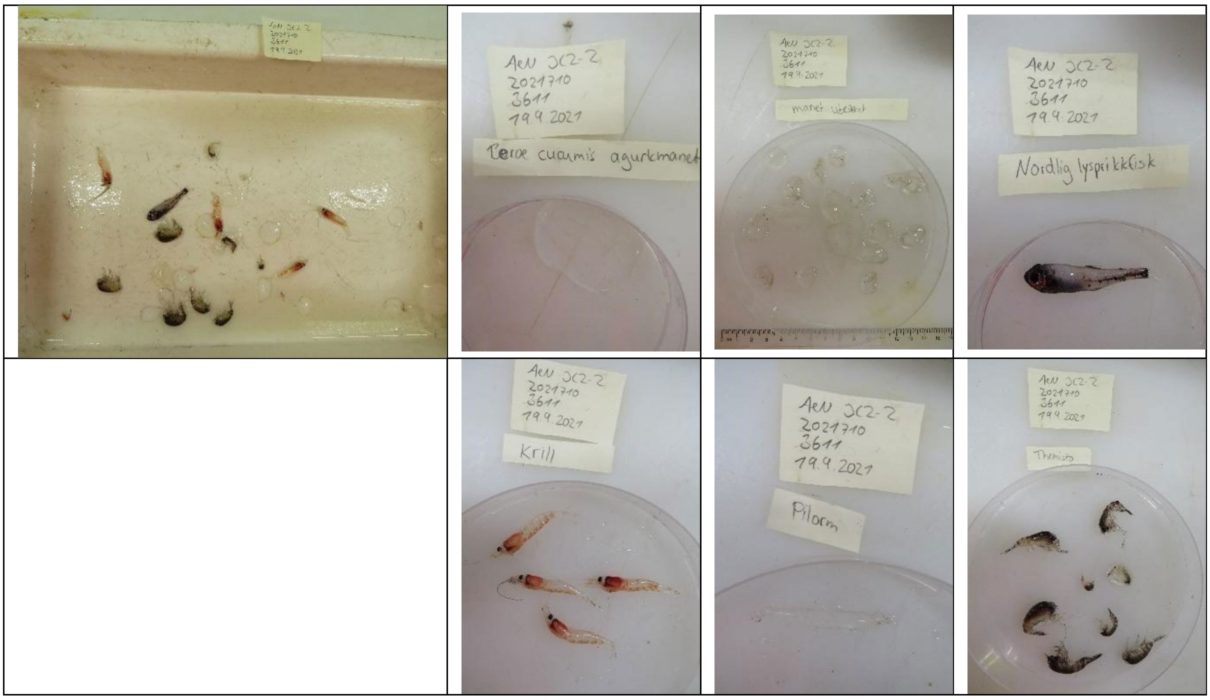

Figure S11. Photos of the catch from AM1m (NLEG39) 3611 (Harstad trawl with fishing depth 460-300 m).

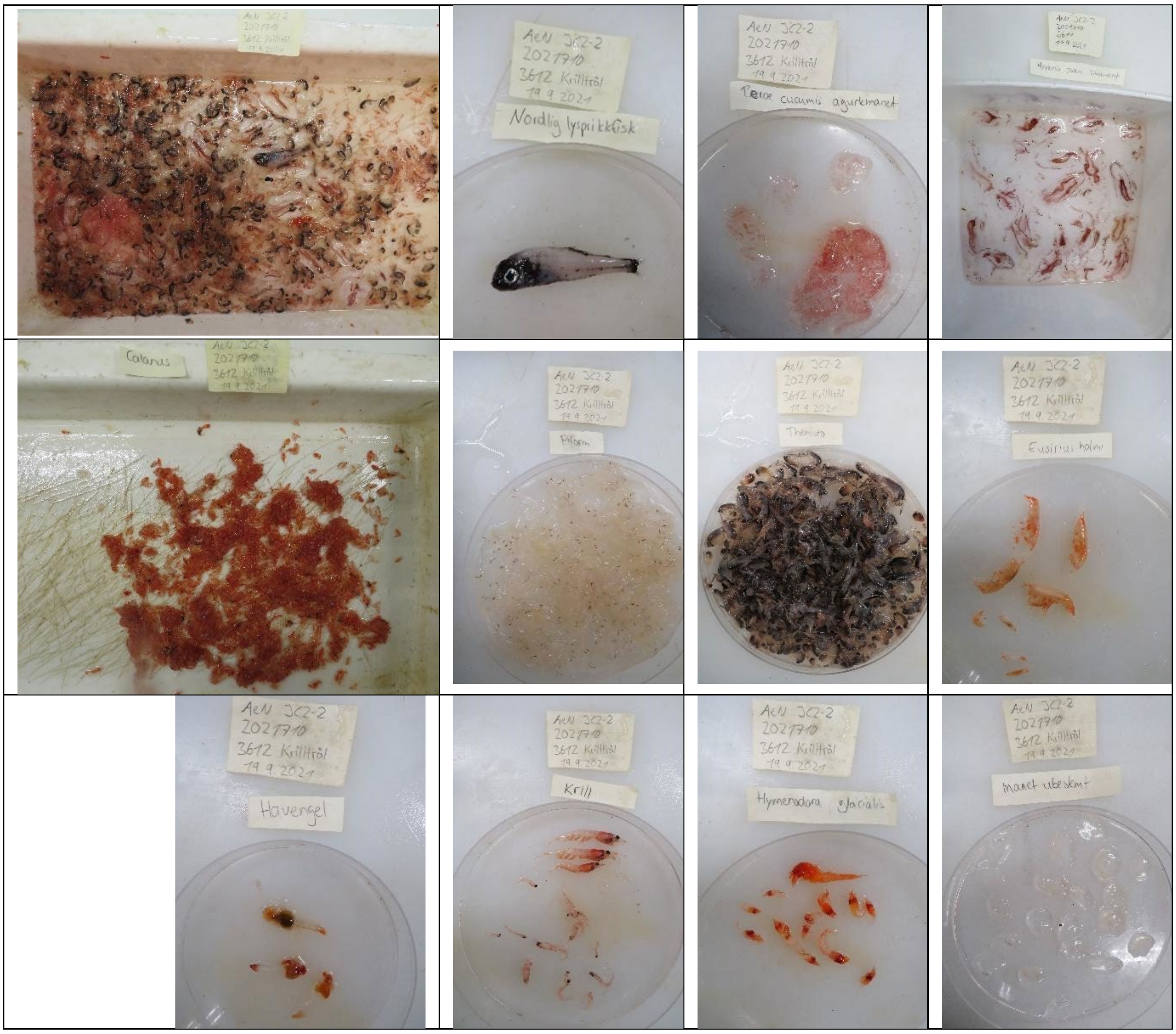

Figure S12. Photos of the catch from AB1mMT (NLEG39) 3612 (macrozooplankton trawl with fishing depth 430-260 m).

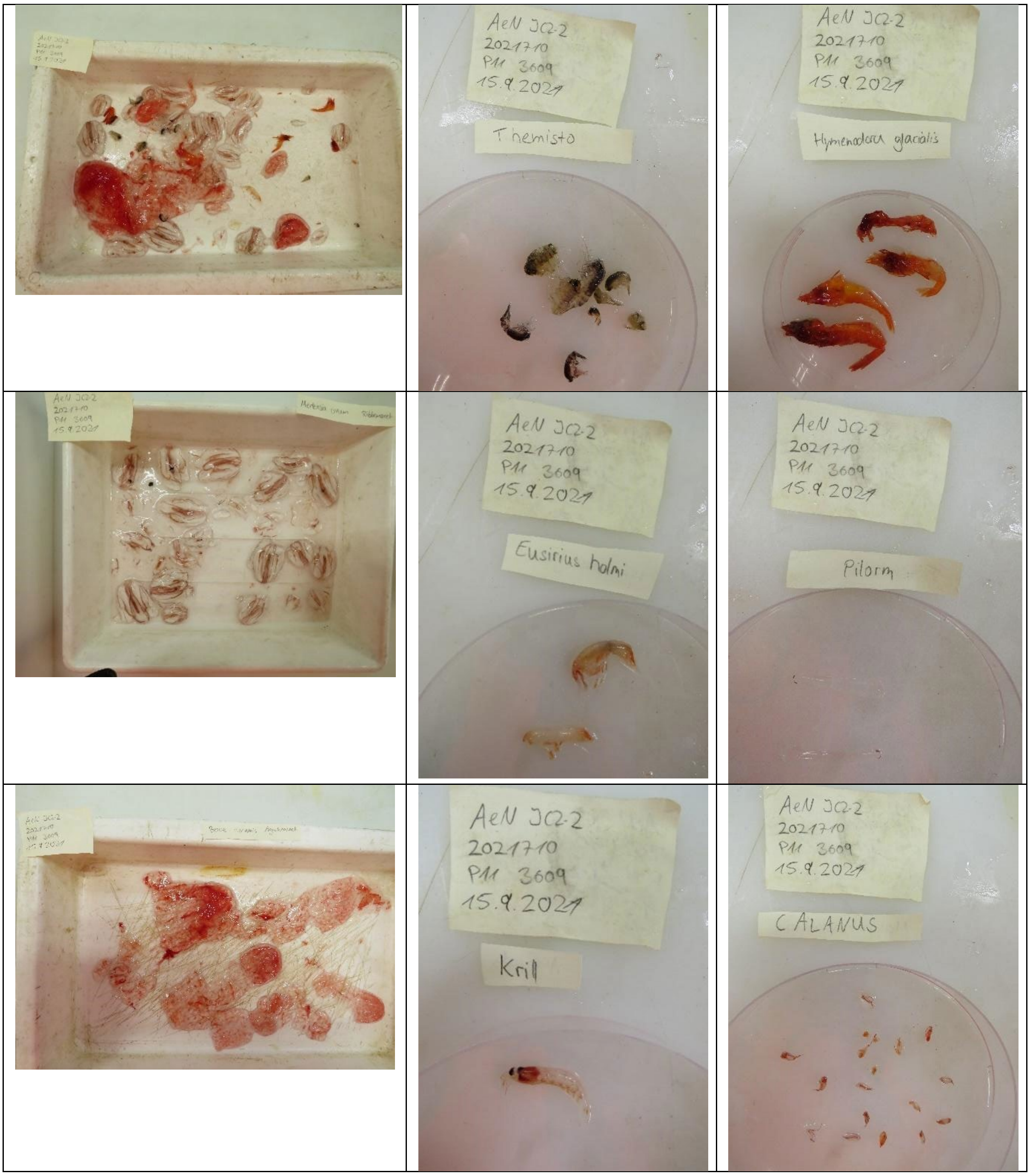

Figure S13. Photos of the catch from AM2em (P11a) 3609 (Harstad trawl with fishing depth 46-484-46 m).

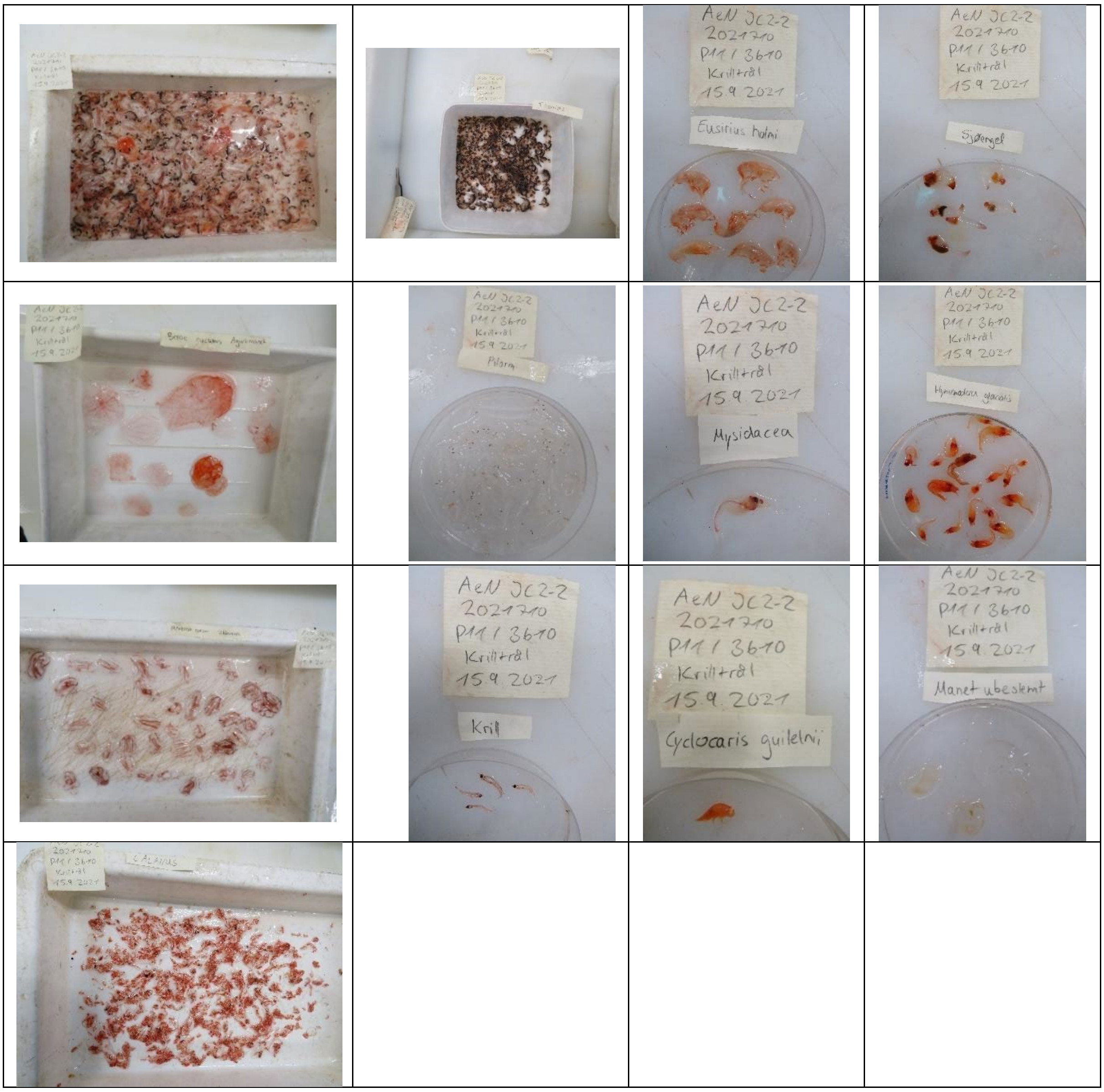

Figure S14. Photos of the catch from AB2emMT (P11a) 3610 (macrozooplankton trawl with fishing depth 49-483-49m).

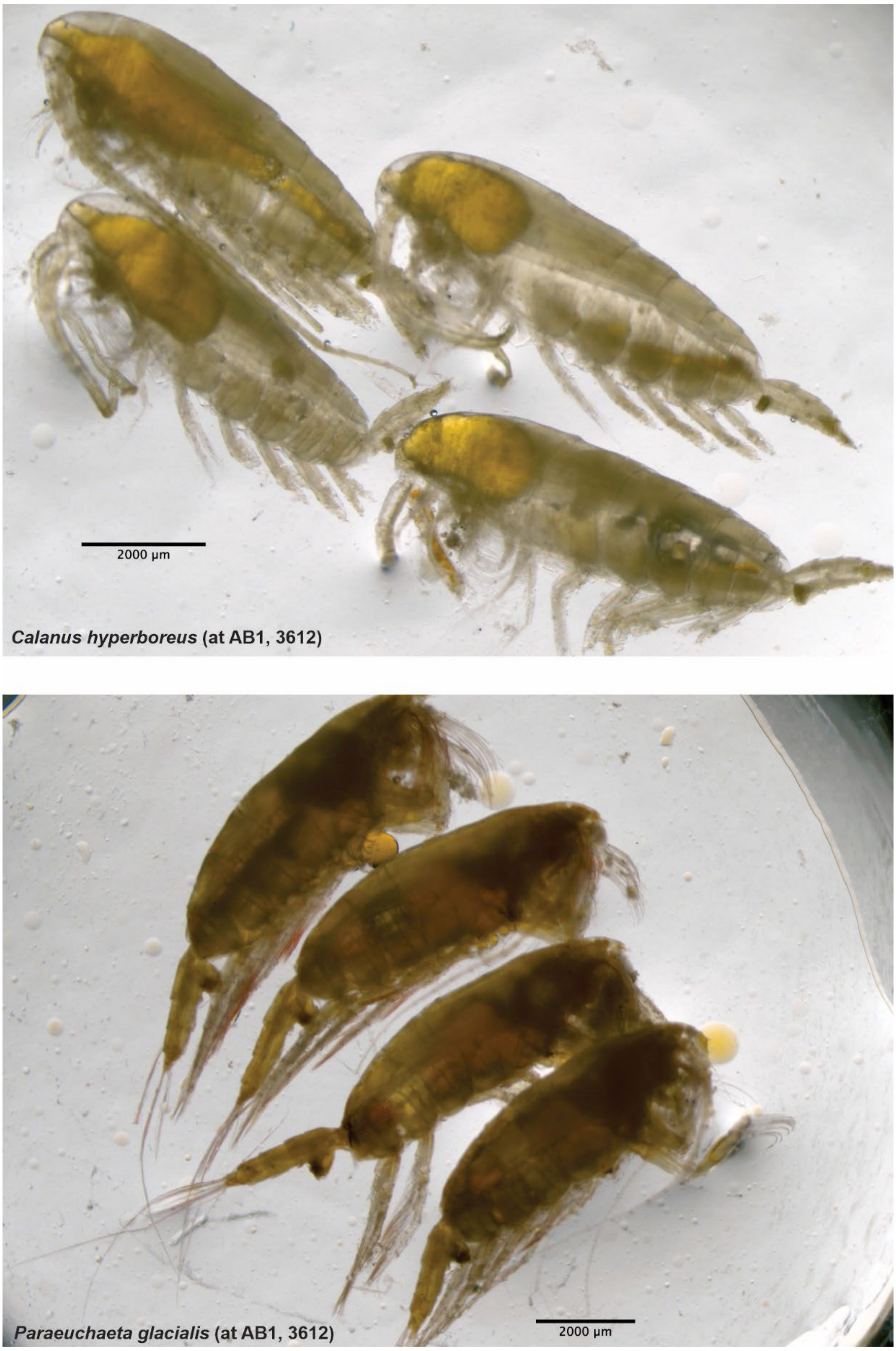

Figure S15. Mature females of *Calanus hyperboreus* (upper) and *Paraeuchaeta glacialis* (lower) from the southern Amundsen Basin (AB1).

Supplementary section 3: Acoustics

Methods and analysis

Acoustical data were obtained from a Simrad EK80 echosounder mounted behind an ice window under the hull. The echosounder was calibrated according to standard procedures[1] at 19.01.2021. Only data from periods when the ship was laying still were used due to mechanical noise when the vessel was moving through sea ice. An hour of acoustic data without noise was selected from as near the trawl stations as possible, and echograms from the six stations are shown in Figs. S16, S18-S21, S23-S24. Applying a threshold of -90 dB gave echosounders data down to at least 500 m for the frequencies 18 and 38 KHz. The usable range was less for the higher frequencies (for 70 KHz about 400 m, for 120 KHz about 200 m, for 200 KHz about 100 m, and for 333 KHz less than 50 m).

The echo integrator threshold in terms of volume backscattering strength (S_V) in dB was set at -90 dB re 1 m^{-1} (ref [2]). The 38 KHz frequency was used to determine backscatter intensity. Analysis was done in the postprocessing tool LSSS v. 2.11.0 (ref [3]) and the backscattering data output were in the form of s_A, Nautical area scattering coefficient (NASC) in standard units (m^2 nmi^{-2} (ref [2])). The data were integrated over the time period and stored at a grid of 10 m depth and 1 hour (3600 seconds).

The analysis of the multifrequency acoustic data included grouping of the Nautical area backscattering coefficients s_A into five classes according to their s_V (definitions and notation of acoustic quantities according to Maclennan[2]) by sequential thresholding at 38 KHz, measurements of relative frequency response (the volume backscattering coefficient s_V at a specific frequency relative to that of a reference frequency), and measurements of target strength (TS) of individual scatterers.

The classification of s_A illustrates how the total backscatter is distributed along an axis from weak scattering (organisms with low TS and/or low density) to strong scattering (organisms with high TS and/or high density). At each location, the s_A over one hour of acoustic data was allocated to five categories of scatterers based on the volume backscattering strength, viz. S70 (all scatterers stronger than -70 dB on 38 KHz), S75 (scatterers in the interval -70 to -75 dB), S80 (scatterers in the interval -75 to -80 dB), S85 (scatterers in the interval -80 to -85 dB) and S90 (scatterers in the interval -85 to -90 dB). The water column was divided into 50 m depth channels, and within each of these channels the s_A was allocated to the various categories by means of sequential thresholding at -85, -80, -75, and -70 dB.

Frequency response gives information about the distribution of various types of scattering organisms since various types of organisms of the same size often have different acoustic properties at different frequencies. The different frequencies were considered down to their acceptable ranges when determining frequency response and we give the relative frequency response between 18 and 38 KHz ($rf_{18/38}$) when feasible.

The TS distribution at a given frequency in a volume of water illustrates the acoustic properties of single targets that give backscatter. High average TS means either a big target and/or a target that gives a strong echo. If for instance fishes with gas filled swim bladders are present, we would expect frequency responses which are highest for the lower frequencies and become lower for higher frequencies, while many planktonic species have an opposite frequency response. A complicating factor is that if gas inclusions are present for instance in siphonophores, they would give frequency responses resembling those from fishes with swim bladder. If big targets are present, we would anticipate TS-values stronger than -40 dB, while small plankton would have TS-values weaker than -80 dB. We mainly considered TS measurement from the 38 KHz, but TS from 18 KHz were also assessed when available. The higher frequencies were only considered when we recorded extraordinary registrations (like at NB1). The TS measurements were identified in LSSS using the following settings: Detector type SED (Single echo detector), Min TS -70 dB, Pulse length determination Level: 6 dB, Minimum echo length: 0.5 (relative to pulse length), Maximum echo length 2.0 (relative to pulse length), Max gain compensation 6 dB, Phase Deviation Check: On. Max phase deviation: 8 steps. Thereafter, we filtered the data restricting them to be within 2 ^{o} off axis to avoid TS distributions being biased towards higher values due to reduced signal-to-noise ratio in the outer parts of the beam.

Nansen Basin

At NB1, the echogram showed scatterers both above 100 m in the epipelagic layer and between 300 and 500 m in the mesopelagic layer (Fig. S16). The epipelagic layer was dominated by organisms with TS_{38} weaker than -55 dB, with number of TS-observations increasing with decreasing TS down to the minimum TS set at -70 dB (Fig. S17a). The frequency response $rf_{18/38}$ was 4.0. The mesopelagic layer showed a TS_{38}-distribution with two modes at -52 and -67 dB (Fig. S17a) and a rather flat $rf_{18/38}$ of 0.7. The catch in the mesopelagic layer consisted of various kinds of gelatinous and crustacean plankton, and three specimens of glacier lanternfish *Benthosema glaciale* with lengths from 4.5 to 5.0 cm. Target strength of the glacier lantern fish is a complicated issue since, contrary to in most swim bladdered fishes, the gas contained in the swim bladder may decrease for increasing fish lengths, and large specimens may totally lack a gas filled swim bladder[4]. Since the TS is mainly determined by

the size of the (gas filled) swim bladder, this may cause large variations in the backscatter properties of different specimens of the same species. Nevertheless, a TS_{38} of -52 dB is considerably stronger than the previous estimates of about -62 dB for adult glacier lanternfish[4]. It is therefore unlikely that most targets detected in this scattering layer are from the species caught in the trawl haul. No larger fish were caught, but one may speculate whether the stronger echoes came from for instance polar cod. If the acoustic targets belonged to that species, the mode at -52 dB would translate into fish length of about 9 cm[5]. However, considering the large pulse volume at the depth of the mesopelagic layer, we cannot rule out that the strongest mode was influenced by multiple targets identified as isolated single targets.

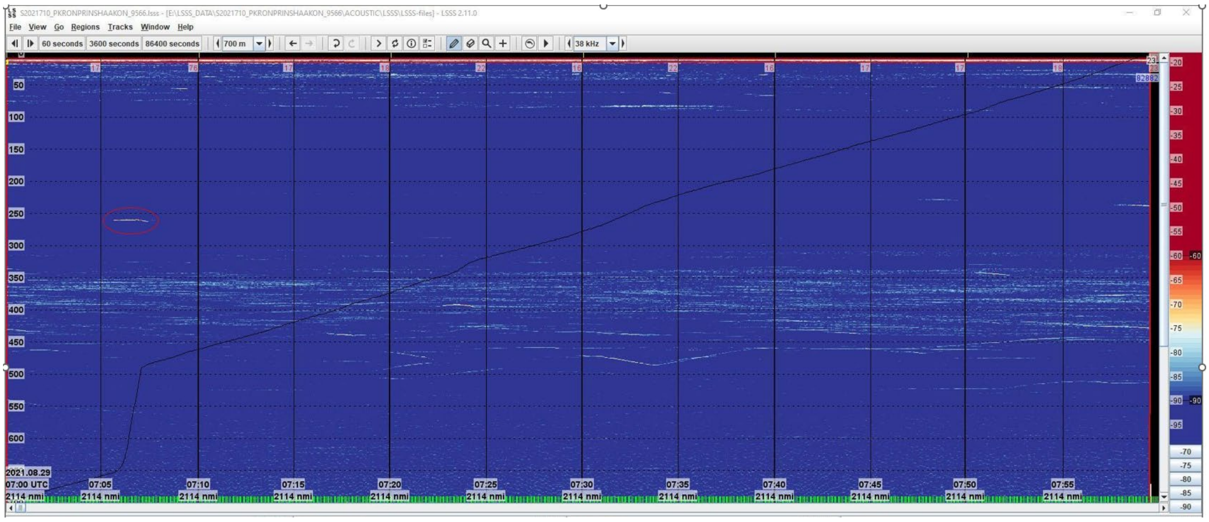

Figure S16. Echogram from location NB1 covering the depth interval 0-700 m and the time period 07:00 to 08:00 on 29th August 2021. The strong echo at 265 m is marked with a red oval.

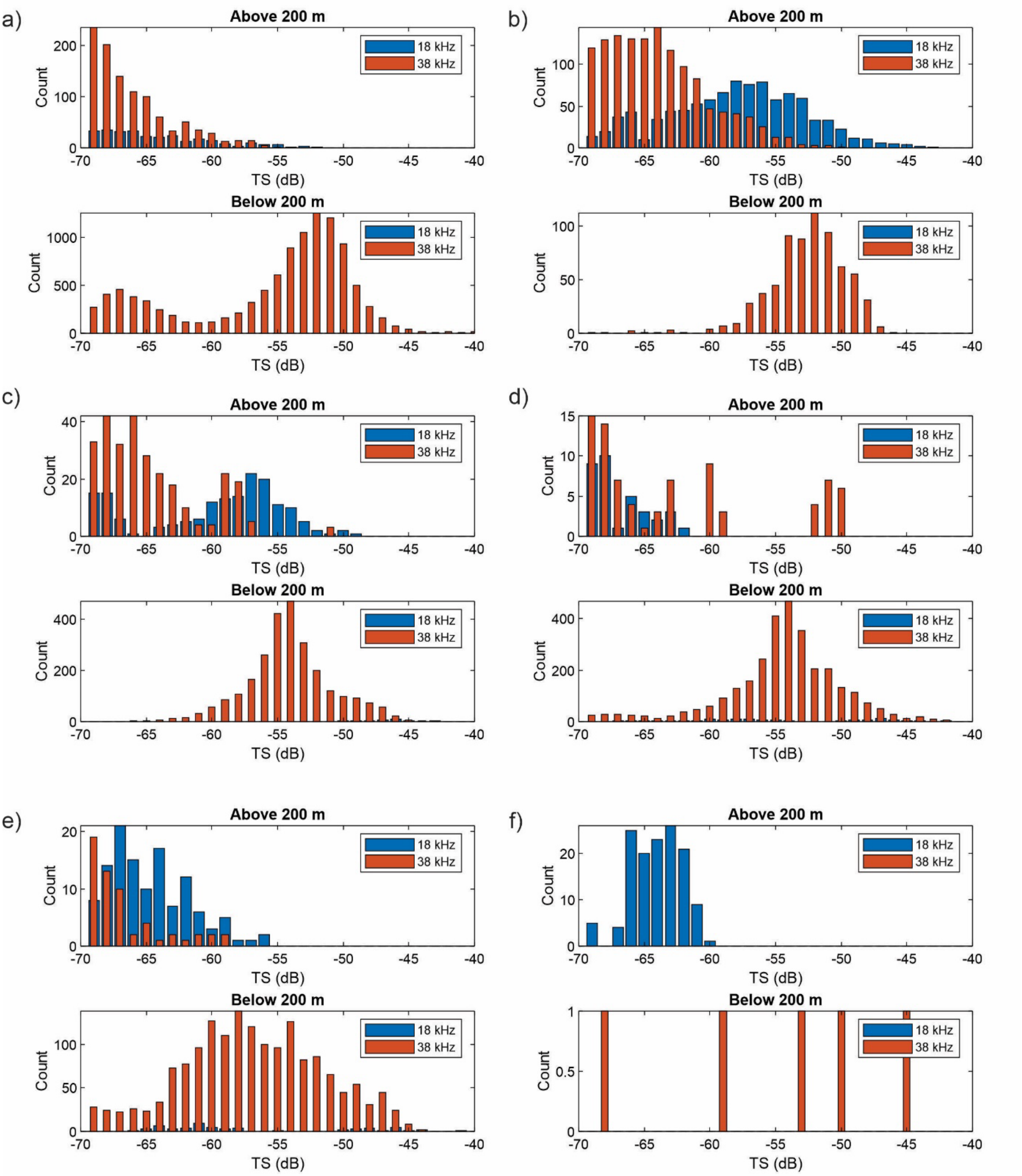

Figure S17. Target strength (TS) at 0-200 m, and below 200 m for each of the six locations (a-f). Only TS data from within the 2 º of the beam axis were included.

At NB1, one single echo from a target at 265 m stood out from the rest with an s_A more than 10 times higher than the maximum s_A in the scattering layers (Fig. S18). This target stayed in the beam for about two minutes, while the vessel speed was between 0.3 and 0.4 knots. More than 20 TS_{38} detections on the target showed maximum values in the range -20 to -32 dB (Fig. S18, lower panels). The maximum TS_{38} observed of -20 dB could possibly stem from a

large Atlantic cod and the mean TS_{38} over the whole track of about -26 dB would translate into a cod of length 130-140 cm. However, the two characteristics of the acoustic signal do not fit closely with what would be expected from a large cod. First, the frequency response curve (Fig. S18, lower panels) is relatively flat from 18 to 120 KHz while a decreasing signal with increasing frequency would be anticipated from a swim bladdered fish like cod. Second, the echo length of this target is longer than the pulse length about 0.75 m, indicating that the target had a vertical dimension exceeding this and thus "stretching" the echo compared to the incident sound pulse. Other possible targets could be a sea mammal or a big fish without a swim bladder. Seals and whales exhale most of the air in the lungs before diving, so the flat frequency response would fit, but it is not very likely that a sea mammal would stay almost motionless for more than two minutes in the water, as is the case here. A more probable candidate is a Greenland shark (*Somniosus microcephalus)*. According to Ona and Nielsen[6], these fishes, which are likely to be found in this area, have acoustic characteristics that agree well with those observed here, and are also known to be very sluggish fishes. Ona and Nielsen[6] measured swimming speeds of from 0.16 to 0.84 ms^{-1} for 15 free swimming Greenland sharks north of Svalbard.

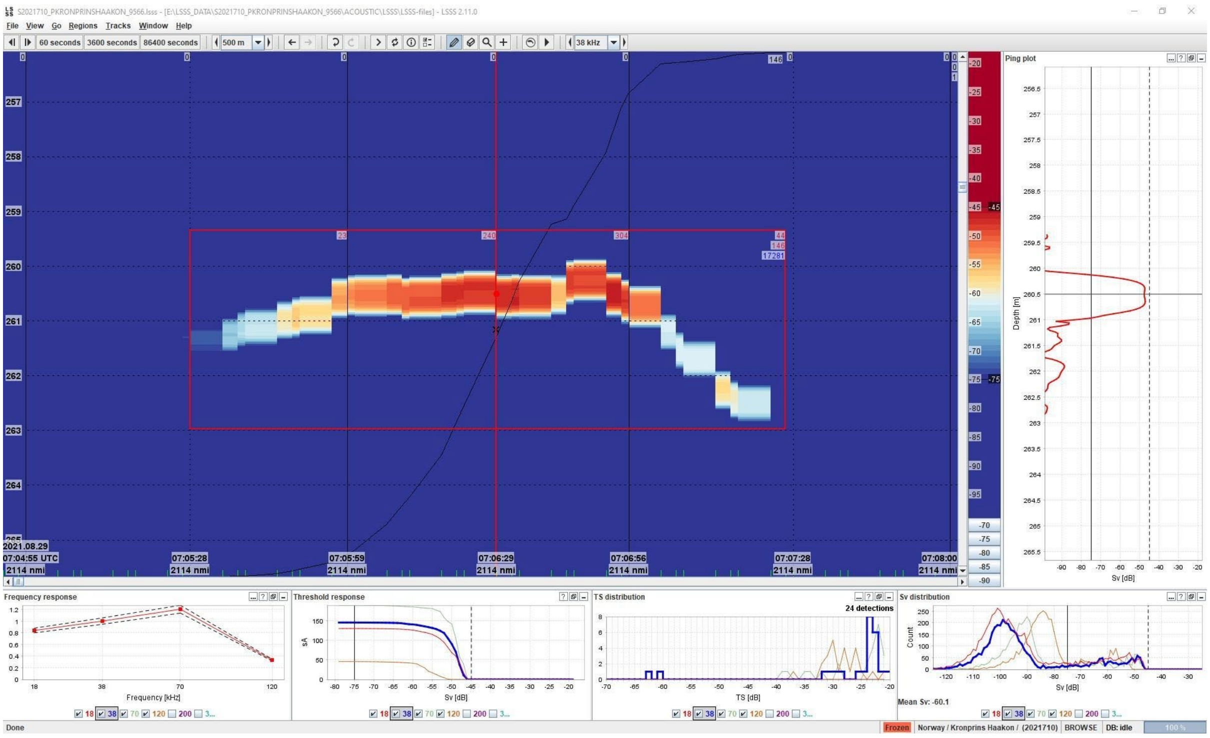

Figure S18. Echogram showing the single strong target at 265 m depth (marked with a red oval in Supplementary figure S16) recorded at 07:06 on the 29th of August 2021. The lower panels show frequency response, threshold response, TS distribution and Sv distribution.

In the central Nansen Basin (NB2), faint echoes forming a scattering layer were present in the depth interval 100-200 m (Fig. S19). Very cold water (-1.5 °C > T > -1.8 °C) occupied the upper 100 m and the scattering appeared at depths where the temperature approached 0 °C (Fig. 4b). The targets located at about 150 m depth were much stronger on 18 than on 38 KHz ($rf_{18/38}$ = ~7) suggesting the presence of organisms with gas inclusions. All targets had TS_{38}-detections weaker than -50 dB (Fig. S17b), indicating that no larger fish were present in the layer. Consistently, the Harstad trawl catches from 150 m depth consisted of a mixture of gelatinous and crustacean plankton and an armhook squid (Table S2). Additionally, one larval Greenland halibut (4 cm long) was caught, which would have TS_{38} far lower than -50 dB since it is without swim bladder. At depths deeper than 200 m the TS_{38}-distribution showed values from -46 dB to -69 dB, peaking at -52 dB (Fig. S17b). Such a distribution would suggest small fish, but fish with swim bladders would have larger $rf_{18/38}$ than observed, and fishes without swim bladder (for instance some species of mesopelagic fishes) would probably have weaker TS_{38}. Thus, it is unclear which organisms which caused this backscatter. At these depths, gelatinous plankton *(Periphylla periphylla)* dominated the trawl catches (by weight) (Table S2).

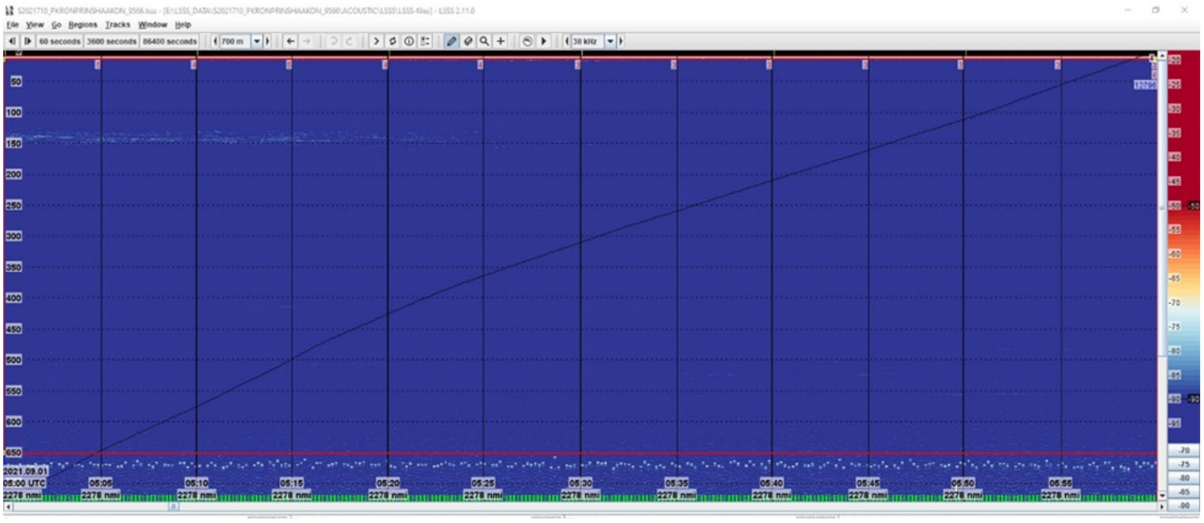

Figure S19. Echogram from location NB2 covering the depth interval 0-700 m and the time period 06:00 to 07:00 on 1st September 2021.

Gakkel Ridge

Faint echoes could be seen at GR1 in a narrow band near the surface and at 400-500 m depth (Fig. S20). Some TS_{38} and TS_{18}-detections with strength from -70 to -49 dB were seen in the upper 200 m (Fig. S17c), where the $rf_{18/38}$ was about 5.0. At greater depths, the TS_{38}-distribution peaked at about -54 dB and the $rf_{18/38}$ was 1.2.

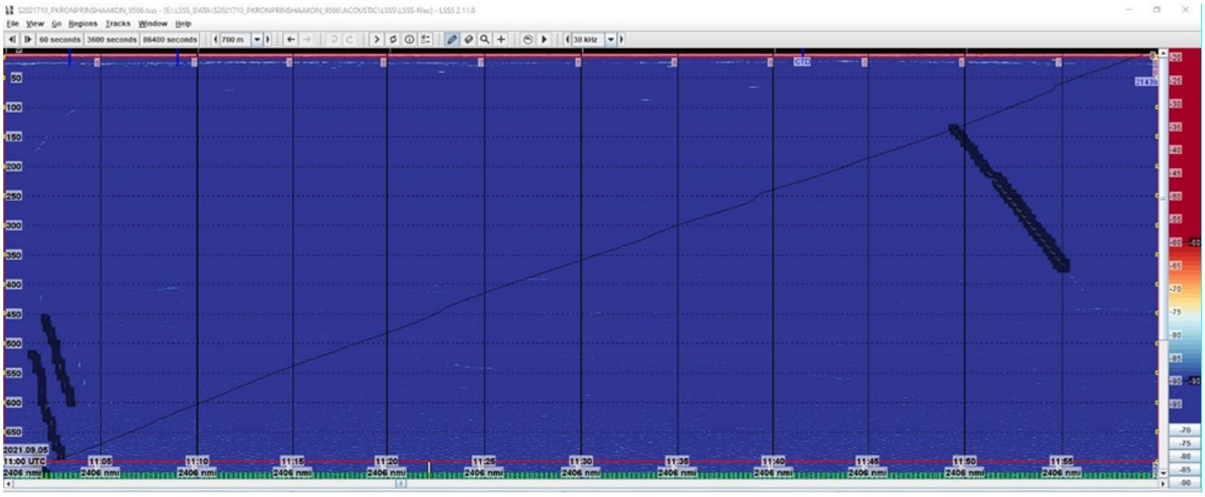

Figure S20. Echogram from location GR1 covering the depth interval 0-700 m and the time period 11:00 to 12:00 on 5th September 2021.

The acoustic registrations at GR2 consisted of two faint scattering layers; one near the surface and one at 300-500 m (Fig. S21 and S22). The upper layer was characterised by a $rf_{18/38}$ of 2.7 and a TS_{38} distribution with increased numbers of observations towards the lower threshold of -70 dB (Fig. S17d). We observed no TS observations stronger than -50 dB (Fig. S17d). The deeper scattering layer had TS_{38} values in the range -42 to -70 dB peaking at -54 dB. The $rf_{18/38}$ was 0.4 in this layer.

A sharp peak of increased fluorescence in the upper water column overlapped with the increased acoustic scattering at GR1 and 2 (Fig. S22), implying that the scattering stemmed from zooplankton gathered in a layer of phytoplankton. However, it is also possible that some of the backscatter originated from the sound velocity contrast in this pycnocline (Fig. S22). The S75 and S80 categories at GR2 were higher than anywhere else in the section, implying a different zooplankton composition or density.

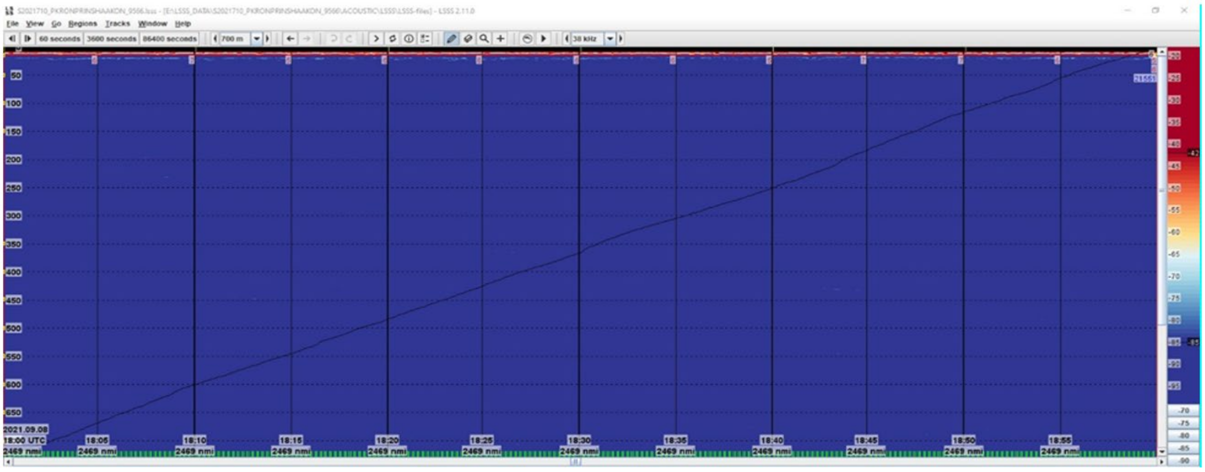

Figure S21. Echogram from location GR2 covering the depth interval 0-700 m and the time period 18:00 to 19:00 on 1st September 2021.

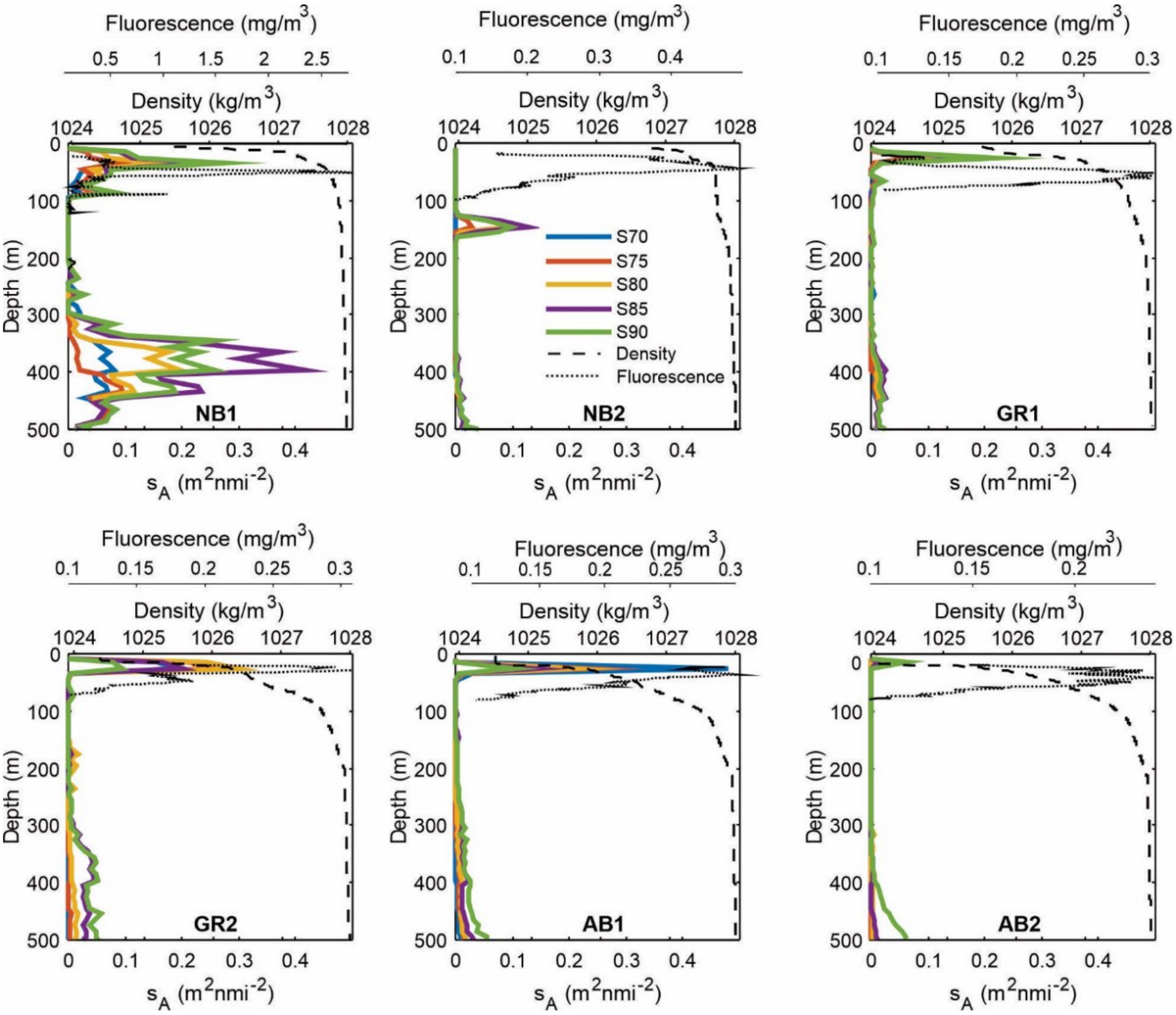

Figure 22. Vertical profiles of s_A by depth at the six locations. Colours show the five categories of scatterers, --- shows density and ...... show fluorescence. The anomalously strong echo from a single target at NB1 was removed before plotting.

Amundsen Basin

At AB1, the echogram showed practically nothing, except for some scattering very close to the surface (Fig. S23). However, the 25-30 m depth layer had a stronger acoustic signal than elsewhere in the study area and appeared to have a different composition and/or density (a stronger S70 fraction) (Fig. S22). As for the Gakkel Ridge, it is unclear if this scattering stemmed from biological organisms or from the pycnocline, which was very steep at this location (Fig. S22). However, the elevated scattering also coincided with a conspicuous peak in fluorescens and may be associated with zooplankton feeding on phytoplankton there. The TS_{38} and TS_{18} observations in the epipelagic layer indicated mostly small organisms (Fig. S17e). The TS_{38} registrations at deeper water resembled those at other locations, but with a wider distribution peaking at -58 dB (Fig. S17e). However, also at this location the TS_{38} distribution at depth revealed numerous TS_{38} registrations at -54 dB.

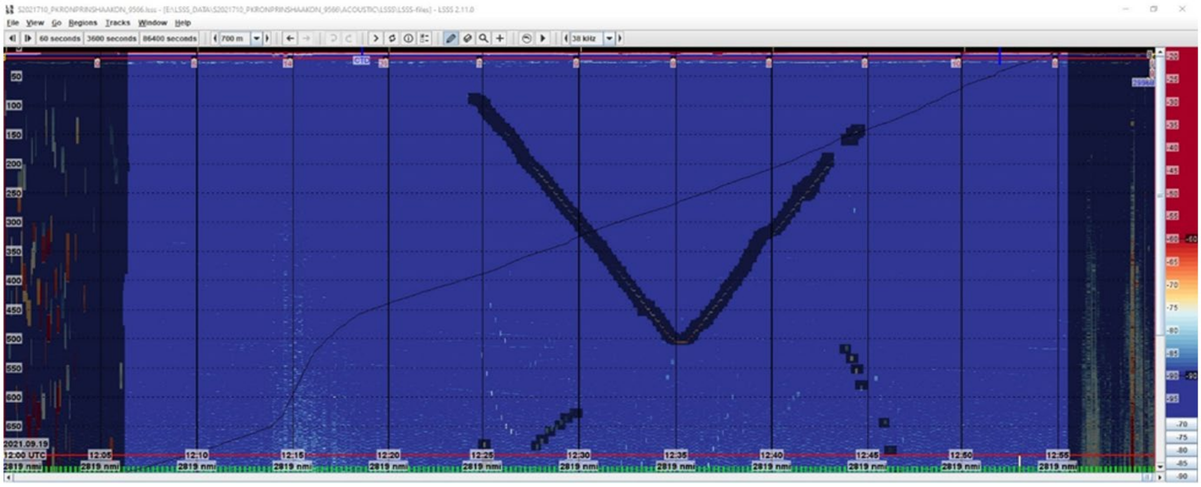

Figure S23. Echogram from location AB1 covering the depth interval 0-700 m and the time period 12:00 to 13:00 on 19th September 2021.

At AB2, a narrow band at 25-35 m depth was visible in the echogram (Fig. S24). The upper water masses were characterised by weak echoes and only the 18 KHz returned TS observations (Fig. S17f). At deeper water there were too few echoes to return TS measurements (Fig. S17f).

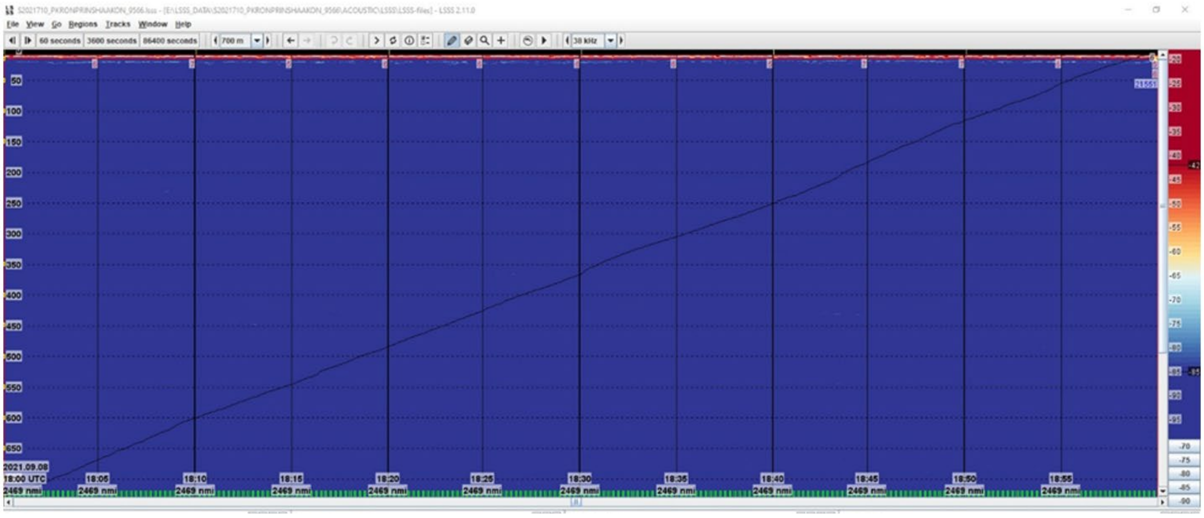

Figure S24. Echogram from location AB2 covering the depth interval 0-700 m and the time period 18:00 to 19:00 on 8th September 2021.

References

1 Foote, K. G. Maintaining precision calibrations with optimal copper spheres. *J. Acoust. Soc. Am.* **73**, 1054-1063 (1983).

2 Maclennan, D. N., Fernandes, P. G. & Dalen, J. A consistent approach to definitions and symbols in fisheries acoustics. *ICES J. Mar. Sci.* **59**, 365-369 (2002).

3 Korneliussen, R. J. *et al.* Acoustic identification of marine species using a feature library. *Methods Oceanogr.* **17**, 187-205 (2016).

4 Scoulding, B., Chu, D., Ona, E. & Fernandes, P. G. Target strengths of two abundant mesopelagic fish species. *The Journal of the Acoustical Society of America* **137**, 989-1000 (2015).

5 Gjøsæter, H. & Ushakov, N. G. Acoustic estimates of the Barents Sea Arctic cod Stock (*Boreogadus saida*). Forage Fishes in Marine Ecosystems. Alaska Sea Grant Collage Program, University of Alaska Fairbanks, 97:01, 485-504 (1997).

6 Ona, E. & Nielsen, J. Acoustic detection of the Greenland shark (Somniosus microcephalus) using multifrequency split beam echosounder in Svalbard waters. *Prog. Oceanogr.* **206**, 102842 (2022).
